# Dual thick and thin filament linked regulation of stretch- and L-NAME-induced tone in young and senescent murine basilar artery

**DOI:** 10.3389/fphys.2023.1099278

**Published:** 2023-03-28

**Authors:** Lubomir T. Lubomirov, Mechthild M. Schroeter, Veronika Hasse, Marina Frohn, Doris Metzler, Maria Bust, Galyna Pryymachuk, Jürgen Hescheler, Olaf Grisk, Joseph M. Chalovich, Neil R. Smyth, Gabriele Pfitzer, Symeon Papadopoulos

**Affiliations:** ^1^ Center of Physiology, Institute of Vegetative Physiology, University of Cologne, Cologne, Germany; ^2^ Institute of Physiology, Brandenburg Medical School Theodor Fontane, Neuruppin, Germany; ^3^ Research Cluster, Molecular Mechanisms of Cardiovascular Diseases, Brandenburg Medical School Theodor Fontane, Neuruppin, Germany; ^4^ Center of Physiology, Institute of Neurophysiology, University of Cologne, Cologne, Germany; ^5^ Institute of Anatomy, University of Cologne, Cologne, Germany; ^6^ Institute of Anatomy, Brandenburg Medical School Theodor Fontane, Neuruppin, Germany; ^7^ Department of Biochemistry and Molecular Biology, Brody School of Medicine at East Carolina University, Greenville, NC, United States; ^8^ Biological Sciences, Southampton General Hospital, University of Southampton, Southampton, United Kingdom

**Keywords:** stretch-induced tone, senescence, basilar artery, non-muscle myosin, caldesmon

## Abstract

Stretch-induced vascular tone is an important element of autoregulatory adaptation of cerebral vasculature to maintain cerebral flow constant despite changes in perfusion pressure. Little is known as to the regulation of tone in senescent basilar arteries. We tested the hypothesis, that thin filament mechanisms in addition to smooth muscle myosin-II regulatory-light-chain-(MLC_20_)-phosphorylation and non-muscle-myosin-II, contribute to regulation of stretch-induced tone. In young BAs (y-BAs) mechanical stretch does not lead to spontaneous tone generation. Stretch-induced tone in y-BAs appeared only after inhibition of NO-release by L-NAME and was fully prevented by treatment with 3 μmol/L RhoA-kinase (ROK) inhibitor Y27632. L-NAME-induced tone was reduced in y-BAs from heterozygous mice carrying a point mutation of the targeting-subunit of the myosin phosphatase, MYPT1 at threonine696 (MYPT1-T696A/+). In y-BAs, MYPT1-T696A-mutation also blunted the ability of L-NAME to increase MLC_20_-phosphorylation. In contrast, senescent BAs (s-BAs; >24 months) developed stable spontaneous stretch-induced tone and pharmacological inhibition of NO-release by L-NAME led to an additive effect. In s-BAs the MYPT1-T696A mutation also blunted MLC_20_-phosphorylation, but did not prevent development of stretch-induced tone. In s-BAs from both lines, Y27632 completely abolished stretch- and L-NAME-induced tone. In s-BAs phosphorylation of non-muscle-myosin-S1943 and PAK1-T423, shown to be down-stream effectors of ROK was also reduced by Y27632 treatment. Stretch- and L-NAME tone were inhibited by inhibition of non-muscle myosin (NM-myosin) by blebbistatin. We also tested whether the substrate of PAK1 the thin-filament associated protein, caldesmon is involved in the regulation of stretch-induced tone in advanced age. BAs obtained from heterozygotes Cald1^+/−^ mice generated stretch-induced tone already at an age of 20–21 months old BAs (o-BA). The magnitude of stretch-induced tone in Cald1^+/−^ o-BAs was similar to that in s-BA. In addition, truncation of caldesmon myosin binding Exon2 (CaD-▵Ex2^−/−^) did not accelerate stretch-induced tone. Our study indicates that in senescent cerebral vessels, mechanisms distinct from MLC_20_ phosphorylation contribute to regulation of tone in the absence of a contractile agonist. While in y-and o-BA the canonical pathways, i.e., inhibition of MLCP by ROK and increase in pMLC_20_, predominate, tone regulation in senescence involves ROK regulated mechanisms, involving non-muscle-myosin and thin filament linked mechanisms involving caldesmon.

## Introduction

We have recently shown that aging of cerebral arteries is associated with a hypercontractile state of smooth muscle (Lubomirov et al., 2017). The hypercontractile state was blunted by NO released from the endothelium and was associated with increased inhibitory phosphorylation of MYPT1 and increased filamentous F-actin protein content. However, the mechanism leading to this hypercontractile response was undetermined as there are many potential sites at which aging might act.

Vascular smooth muscle tone is primarily the result of the cyclic interaction of thick myosin filaments with filamentous actin (F-actin) in a cycle driven by ATP hydrolysis (Arner and Pfitzer, 1999). Although smooth muscle myosin II is the primary motor protein of smooth muscle contraction, there is increasing evidence that non-muscle myosin II is involved in slowly developing contractions (Morano et al., 2000) and tension maintenance ((Zhang and Gunst, 2017) reviewed in (Brozovich et al., 2016)). Regulation of smooth muscle contraction occurs by altering the level of phosphorylation of myosin, by altering the extent of actin polymerization into thin filaments, and by proteins that bind directly to actin, such as caldesmon (Morgan and Gangopadhyay, 2001). All of these points of regulation are potentially altered by aging leading to the hypercontractile state.

Both myosin types, smooth muscle and non-muscle myosin II, are activated by phosphorylation of regulatory light chains (MLC_20_) on serine19 (S19) (Zhang and Gunst, 2017). Phosphorylation is catalyzed by Ca^2+^-calmodulin dependent myosin light chain kinase (MLCK) in response to an increase in cytosolic [Ca^2+^]. The rise in Ca^2+^ is triggered by various extracellular stimuli such as neurotransmitters, metabolites and mechanical forces (Brozovich et al., 2016). MLC_20_ is phosphorylated additionally by Ca^2+^-independent, non-canonical MLC-kinases, which include RhoA-kinase (ROK) (Amano et al., 1996).

Dephosphorylation of MLC_20_ is catalyzed by a constitutively active type 1 phosphatase (MLCP) the activity of which is modulated by phosphorylation of its myosin targeting (regulatory) subunit MYPT1 at several serine and threonine residues (Matsumura and Hartshorne, 2008). This allows force to increase or decrease without changes in cytosolic [Ca^2+^], a phenomenon known as Ca^2+^-sensitization and desensitization, respectively [reviewed in (Somlyo and Somlyo, 2003)].

The RhoA-ROK kinase pathway is the key mechanism of increasing Ca^2+^-sensitivity by phosphorylation of MYPT1 at threonine-696 and threonine-853 (Matsumura and Hartshorne, 2008; Puetz et al., 2009). The nitric oxide (NO) protein kinase G (PKG) signaling cascade decreases Ca^2+^-sensitivity by antagonizing Rho-PKG signaling, which disinhibits MLCP by dephosphorylation of MYPT1 (Puetz et al., 2009) and/or activation of MLCP by phosphorylation of MYPT1 at the PKG site serine-668 (Yuen et al., 2011).

Force is also diminished when actin filaments are disassembled or depolymerized. RhoA and ROK promote adhesome complex formation and polymerization of a small G-actin pool at the cell cortex upon contractile stimulation (Zhang and Gunst, 2019). Large actin filaments are anchored to the cell membrane *via* adhesion proteins, which contain some cortical actin polymers. They allow force to be transmitted from myofilaments to the extracellular matrix and act independently of pathways that activate cross-bridge cycling (Gunst and Zhang, 2008). Interfering with actin polymerization results in diminished active force (Zhang et al., 2018). Cortical actin polymerization induced by biomechanical stimulation and contractile agonists requires recruitment of inactive adhesome proteins to membrane adhesion junctions, where they are activated (Gunst and Zhang, 2008; Zhang et al., 2015). The underlying complex signaling mechanisms have been delineated in detail in airway smooth muscle [reviewed in (Gunst and Zhang, 2008)]. They involve the small GTPase, RhoA upstream of non-muscle myosin II (Zhang and Gunst, 2017), and ROK upstream of p21-activated protein kinase (PAK) (Zhang et al., 2018), and eventual activation of the N-WASp and Arp2/3 complex.

A growing body of evidence supports the notion that F-actin dynamics is also involved in regulation of vascular smooth muscle tone (Yamin and Morgan, 2012) and specifically in the myogenic responses of rat brain vasculature (Cipolla et al., 2002; Moreno-Dominguez et al., 2013; Walsh and Cole, 2013). Moreover, an increased F-actin content was associated with a hypercontractile state of old murine brain arteries (Lubomirov et al., 2017).

PAK1 is not only an upstream activator of actin polymerization (Zhang et al., 2018), but also a stimulator of force production by phosphorylating caldesmon and reversing its inhibitory effect on actomyosin interaction (Van Eyk et al., 1998). Caldesmon is a myosin-, actin, calmodulin-, and tropomyosin-binding protein, and is expressed in two isoforms generated by alternative splicing [(Ruegg and Pfitzer, 1991; Gusev, 2001) rev. (Pfitzer et al., 1993a)]. The smooth muscle specific isoform, h-CaD, is found in the actomyosin domain of smooth muscle cells, where it presumably tethers actin- and myosin filaments and stabilizes the contractile machinery (Pfitzer et al., 1993b; Smolock et al., 2009; Putz et al., 2021). Both isoforms inhibit actomyosin MgATPase activity and contraction at constant MLC_20_ phosphorylation. CaD may act as a molecular brake of smooth muscle contraction especially at low MLC_20_ phosphorylation and cytosolic [Ca^2+^] (Smolock et al., 2009; Putz et al., 2021). Phosphorylation of caldesmon at several threonine and serine residues by various protein kinases, including PAK1, facilitates activation of MgATPase activity and cross bridge cycling. PAK1 is activated by ROK-dependent phosphorylation (Zhang et al., 2018), giving rise to the interesting hypothesis that ROK may relieve the normal inhibition of tension development caused by caldesmon.

To conclude there is evidence primarily derived from smooth muscle tissue from young animals that contractile activity is regulated in addition to the canonical pathway *via* phosphorylation of MLC_20_ by, actin based mechanisms, i.e., actin filament dynamics and/or the thin filament linked protein caldesmon. In these pathways, ROK is center stage and regulates different signaling cascades that target these mechanisms. Little is currently known about the contribution of these pathways in brain arteries from senescent mice.

In the present work we explored whether changes in basal phosphorylation levels of the Rho-kinase effectors MLCP, non-muscle myosin, as well as actin filament dynamics parallel the augmented in stretch-induced tone in old basilar arteries. Further, we investigated whether ablation of the PAK1 effector, caldesmon augments stretch induced tone. Specifically we tested the following hypotheses: 1) stretch induced tone is augmented in senescent basilar arteries in a ROK-dependent manner, 2) ROK is upregulated in senescence and serves as a central signaling hub, which not only increases inhibitory phosphorylation of MYPT1 but also of non-muscle myosin and PAK1 3) the PAK1-target caldesmon acts as a molecular brake to attenuate stretch induced tension rise.

## Materials and methods

Ethic statement and detail description of all materials and methods can be found in the online supplement.

## Results

Three different types of muscle tone were examined in this work: 1) stretch-induced tone refers to tone that is present after stretching y-BA to IC90 (90% of the internal circumference at wall tension corresponding to a transmural pressure of 100 mmHg (IC90); see expandet “Methods” section in online supplement) and in s-BA it refers to the slow tension rise that develops in 20–25 min after stretching the preparations to IC90 in the absence of treatment. This tone is maintained throughout the experiment. 2) L-NAME-induced tone develops in response to addition of L-NAME, a pan-NOS inhibitor; 3) agonist-induced tone occurs after stimulation with contractile agonist, U46619. Our first goal was to determine the effect of ROK on each of these types of muscle tone.

### ROK inhibition blunts stretch-, L-NAME-, and agonist-induced tone in young murine basilar arteries

**FIGURE 1:**
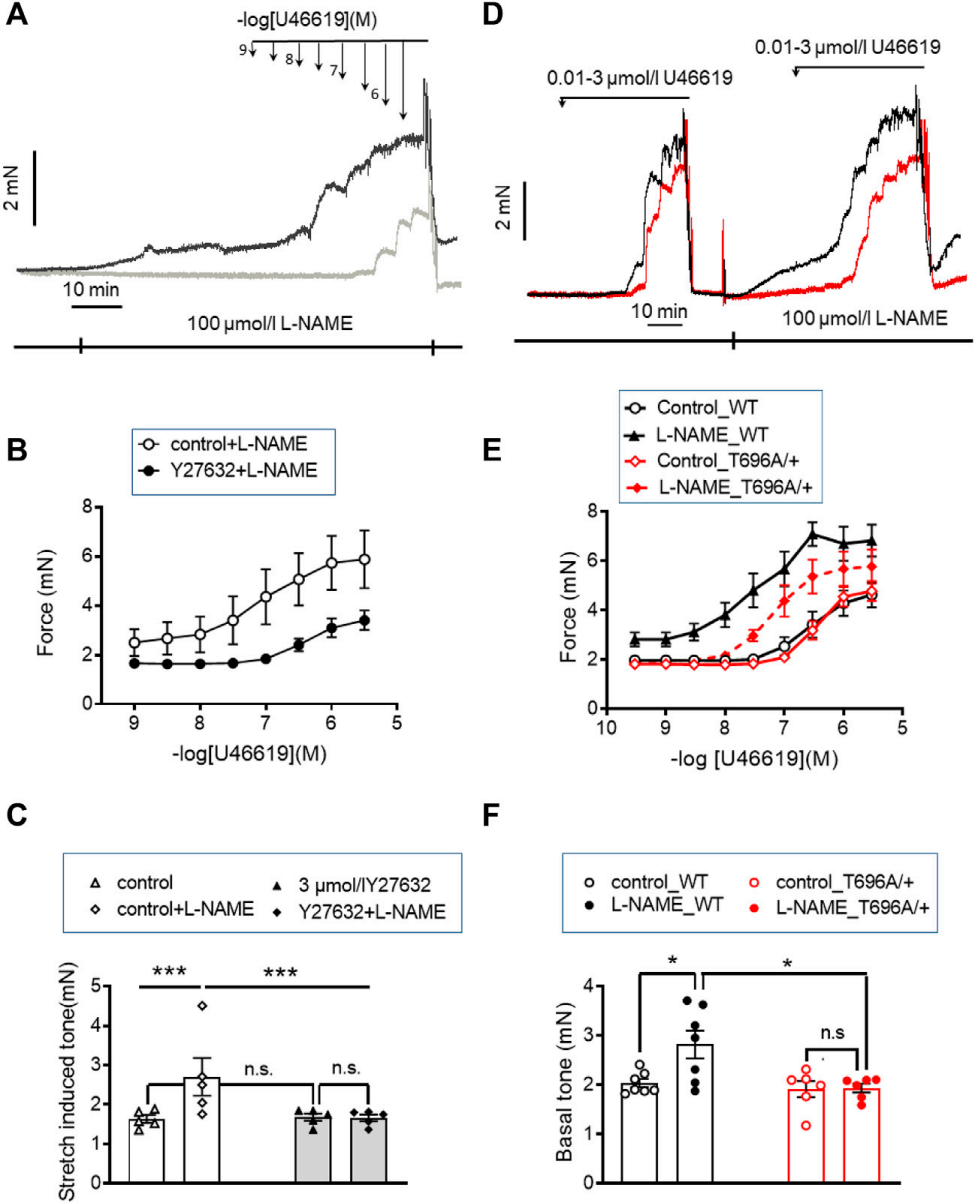
Stretch-, L-NAME-, and agonist-induced force in young murine basilar arteries obtained from WT and MYPT1-T696A/+ -mice. **(A–C)** Original force tracings and statistical summary from measurement of stretch-induced tone stretch-induced tone in y-BAs from WT-animals treated with 100 μmol/L of pan-NOS-inhibitor L-NAME in the presence of 3 μmol/L Y27632 (gray) or vehicle (H_2_O; black). Depicted *p*-values represent results from 5 independent experiments (***p < 0.001; n.s.—not significant, *p* > 0.05; one way ANOVA *n* = 5). **(D–F)** Original force tracings and statistical evaluation of stretch-induced tone, L-NAME- and agonist-induced tone of y-BAs from WT (black) and MYPT1-T696A/+ -mice (red) under control conditions (controls) and after application of 100 μmol/l L-NAME (*n* = 6–7; *p < 0.05; unpaired *t*-test; n.s.—not significant). Bars represent mean ± SEM.

Tone in the absence of L-NAME amounted to ∼1.7 mN, present after the normalization procedure. To demonstrate that this increase in tone involves ROK, ring preparations from BAs were treated after the normalization procedure (20–25 min equilibration in PSS see “Methods”) with 3 μmol/L Y27632 for 10 min, followed by addition of 100 μmol/L of the pan-NOS inhibitor L-NAME for another 20 min in the presence of Y27632. Pretreatment with Y27632 did not alter stretch-induced tone (1.69 ± 0.1 mN in the presence of Y27632 versus 1.73 ± 0.28 in non-treated time matched controls), but prevented the increase in L-NAME induced tone, which was 1.66 ± 0.1 mN in the presence of Y27632 and 2.7 ± 0.5 mN in the absence of Y27632 (Figures 1A, C).

We then tested whether ROK inhibition also attenuated the agonist-induced tone by the thromboxaneA_2_ analogue, U46619. In the presence of Y27632, the cumulative concentration-response relation was shifted to the right; the pEC_50_ value was significantly lower with than without Y27632 in time-matched controls (6.3 ± 0.1 logarithmic units vs. 7.1 ± 0.18; *p* < 0.01; *n* = 5). Maximal force was also lower (F_max_ 3.68 ± 0.37 mN with vs. 5.89 ± 1.3 mN without Y27632, *p* < 0.05; *t*-test; *n* = 5; Figure 1B). However, Y27632 at a concentration of 3 μmol/L (concentration able to nearly completely dephosphorylate ROK-site of targeting subunit of MLCP, MYPT1-T853; Figures 5A, D; Figures 8C, D) attenuated U46619 contractile response by only ∼40% compared to complete inhibition of L-NAME induced tone, suggesting that a part of the U46619-induced tone involves one or more additional mechanisms, distinct from ROK activation.

### Effect of blocking phosphorylation at threonine 696 of MYPT1 on agonist-induced tone in y-BA

The inhibitory effect of the ROK inhibitor on L-NAME and U46619 induced tone may partially result from reduced phosphorylation of MYPT1-T696 (Puetz et al., 2009), a site that is phosphorylated by several protein kinases including ROK. Phosphorylation of this site inhibits MLCP activity *in vitro* (Feng et al., 1999). To test the involvement of phosphorylation this site in L-NAME and U46619 induced contractile activity, we used BAs from 2 months old wild type and heterozygous MYPT1-T696A/+ mice, in which the phosphorylation propensity of MYPT1 at T696 was genetically lowered by mutating the threonine-residue into a non-phosphorylatable alanine (MYPT1-T696A/+).

Ring preparations from MYPT1-T696A/+ and WT BA were stimulated after mounting and equilibration in PSS by cumulatively increasing concentrations of U46619. U46619 induced a concentration dependent rise in tone with similar F_max_ and pEC_50_ values in mutant and WT littermates (*p* > 0.05; *n* = 7–6; Figures 1D–E). After wash-out of the agonist with PSS, the vessels were pre-incubated for 20 min in L-NAME (100 μmol/L), followed by a second U46619 concentration-response relation (Figures 1D, E). In time-matched controls, the second concentration-response relation was obtained in the absence of L-NAME (*n* = 4–5; Supplementary Figure S2). L-NAME shifted the U46619-concentration-response curve to the left in both WT and mutant BA and increased F_max_, whereby neither F_max_ nor the pEC_50_ values differed between MYPT1-T696A/+ and WT littermates (*p* > 0.05; *n* = 7–6; Figures 1D, E). No significant difference between the first and second U46619 concentration response curve was observed in time matched controls (*n* = 5–4; Supplementary Figure S2). L-NAME-induced tone was significantly less in mutant BA than in WT (mutant: 1.95 ± 0.1 mN vs. WT 2.82 ± 0.2 mN, *n* = 7–6; Figures 1D, F). Thus, the effect of the mutation of MYPT1 and Y27632 on L-NAME induced tone were of similar magnitude. All contractile parameters of BAs from MYPT1-T696A/+ and WT mice are given in Table 1.

**TABLE 1:** Contractile parameters of intact preparations from young basilar arteries from wild type (WT) and heterozygous (MYPT1-T696A/+) mice.

Vessel	pEC_50_	ΔpEC_50_ ^a^	F_max_ (mN)	ΔF_max_ (mN)	Stretch- and L-NAME induced tone (mN)	n
WT Control	6.4 ± 0.1***	—	4.5 ± 0.7	—	2.03 ± 0,1	7
WT + L-NAME	7.4 ± 0.1	1.0 ± 0.1	6.8 ± 0.7*	2.4 ± 0.7	2.82 ± 0.3	7
T696A/+ Control)	6.5 ± 0.1^§§§, n.s.^	—	5.3 ± 0.6	—	1.91 ± 0.2	6
T696A/+ L-NAME)	7.2 ± 0.1*	0.6 ± 0.1	5.8 ± 0.7^n.s.^	0.5 ± 0.4	1.95 ± 0.1	6

Results: ***p < 0.0001; pEC_50_ WT Control vs. pEC_50_ WT + L-NAME; 2way ANOVA.

^§§§^ *p* < 0.0001; pEC_50_ T696A/+ Control vs. pEC_50_ T696A/+ L-NAME; 2way ANOVA.

*p < 0.05 pEC_50_ WT + L-NAME vs. pEC_50_ T696A/+ L-NAME; 2way ANOVA.

^n.s.^ *p* > 0.05 pEC_50_ WT Control vs. pEC_50_ T696A/+ Control; 2way ANOVA.

*p = 0.03; F_max_ WT Control vs. F_max_ WT + L-NAME; unpaired *t*-test.

^n.s.^ *p* > 0.05 F_max_ T696A/+ Control vs. F_max_ T696A/+ L-NAME; unpaired *t*-test.

^a^ Experimental pEC_50_—control value.

### L-NAME increased tone and MLC_20_ phosphorylation in WT but not in MYPT1-T696A/+ BA from young mice

**FIGURE 2:**
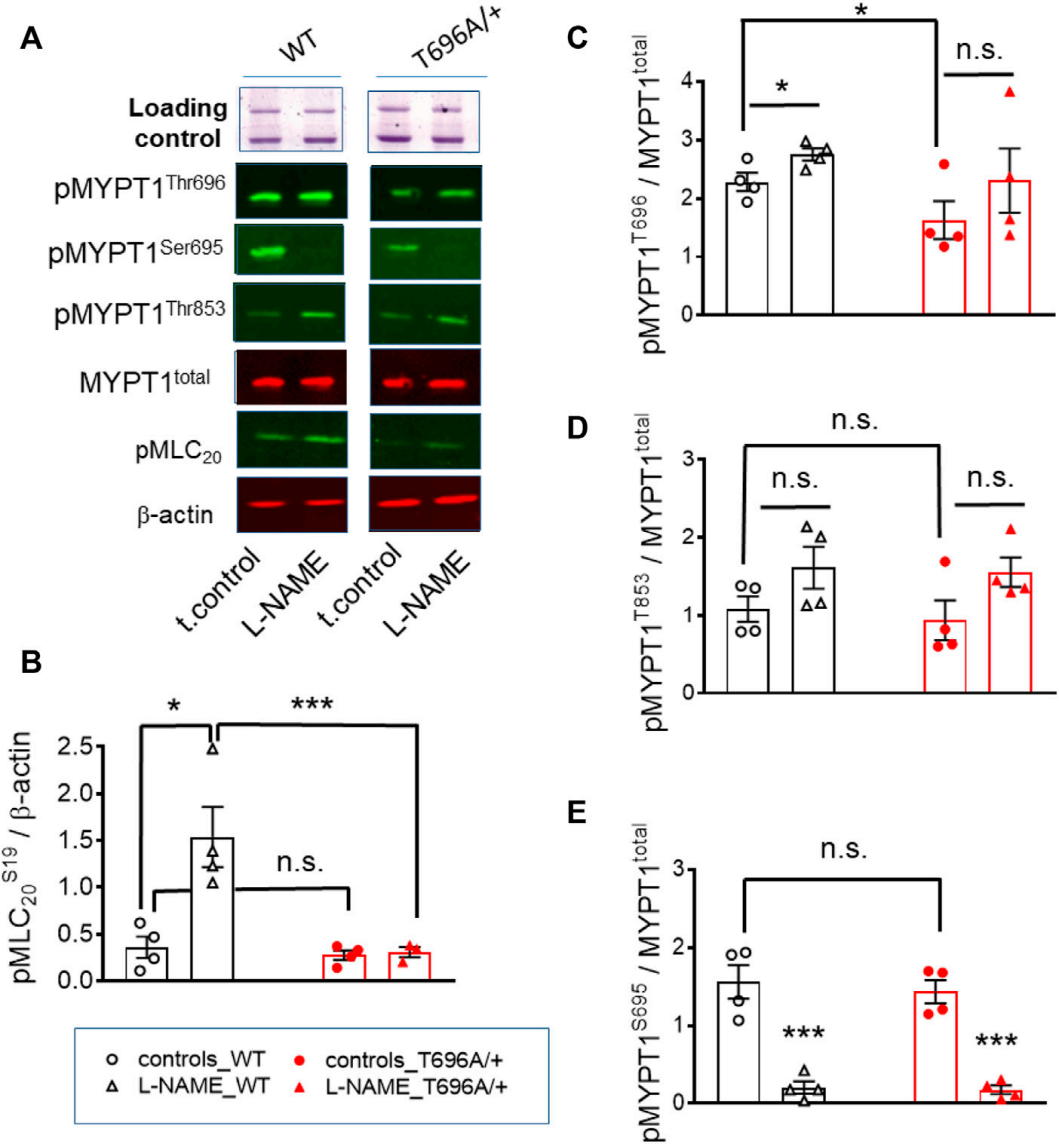
Basal and L-NAME-induced phosphorylation of pMLC_20_-S19, pMYPT1-T696, pMYPT1-T853, pMYPT1-S695 in basilar arteries from young WT and MYPT1-T696A/+ -mice. Original chemiluminograms **(A)** and statistical evaluation **(B–E)** of the immunoreactive signals of lysates of y-BAs from WT and MYPT1-T696A/+ mice transferred on nitrocellulose membranes and probed with antibodies against pMLC_20_-S19, pMYPT1-T696, pMYPT1-T853, and pMYPT1-S695. Statistical figures represent the ratio of the immunoreactive signal of pMLC_20_-S19 normalized to *β*-Actin or pMYPT1-T696, pMYPT1-T853, and pMYPT1-S695 normalized to MYPT1-total (*n* = 4). Arteries were treated using same experimental protocol as in Figure 1. Statistic comparison on **(B)** represents evaluation using two-way ANOVA (***p < 0.0001; *n* = 4). ***p < 0.0001, *p < 0.05 represents evaluation by unpaired *t*-tes*t* (*n* = 4). n.s.—not significant.

Next, we tested in separate experiments whether the reduction in tone induced by L-NAME in y-BAs from MYPT1-T696A/+ mice was related to lower phosphorylation levels of the regulatory light chain of myosin (MLC_20_) under basal conditions. For technical reasons, the vessels were mounted on two wires (ø 25 µm) but not stretched as for mechanical experiments. Basal phosphorylation levels of MLC_20_-S19 in WT and MYPT1-T696A/+ were similar (Figures 2A, B). In line with the attenuated force, incubation with L-NAME was associated with increased MLC_20_-S19 phosphorylation in BA of WT, but not in BA of MYPT1-T696A/+.

We further tested, whether observed differences in MLC_20_-S19 phosphorylation were caused by differences in the phosphorylation of MYPT1 at T696 and T853 and thus in activity of MLCP. Under control conditions, phosphorylation of MYPT1 at T696 (PSS, no treatment) was higher in BA from WT-mice than in those from MYPT1-T696A/+-mice (*p* < 0.05; unpaired *t*-test; *n* = 4; Figures 2A, C). Nominal maximal pMYPT1-T696 phosphorylation was assessed by stimulation with 0.1 μmol/L calyculin, a type1 phosphatase inhibitor (Chen et al., 2015). As expected, MYPT1-T696 phosphorylation in calyculin treated mutant BA amounted to only ∼50% of that in WT BA (Supplementary Figure S3). We did not observe a significant change in phosphorylation of pMYPT1-T853 between WT and mutant mice, under control conditions (PSS, no treatment) or after incubation with L-NAME (Figures 2A, D). This result suggests that this site, which is phosphorylated only by ROK, is not affected by silencing phosphorylation in MYPT1-T696.

To test whether altered PKG activity may contribute to the lower L-NAME induced tone, we determined phosphorylation of MYPT1-S695, which is specifically phosphorylated by PKG. L-NAME decreased phosphorylation of this site, indicated that NO-PKG signaling is constitutively active in this preparation. Phosphorylation of pMYPT1-S695 was similar in WT and mutant BA (Figures 2A, E). These results also indicated that the alanine mutation of T696 did not affect the phosphorylation of the neighboring S695, and further, that the NO-PKG pathway was not affected by the mutation.

### Ca^2+^-sensitivity is lower in young, α-toxin permeabilized MYPT1-T696A/+ BA

**FIGURE 3:**
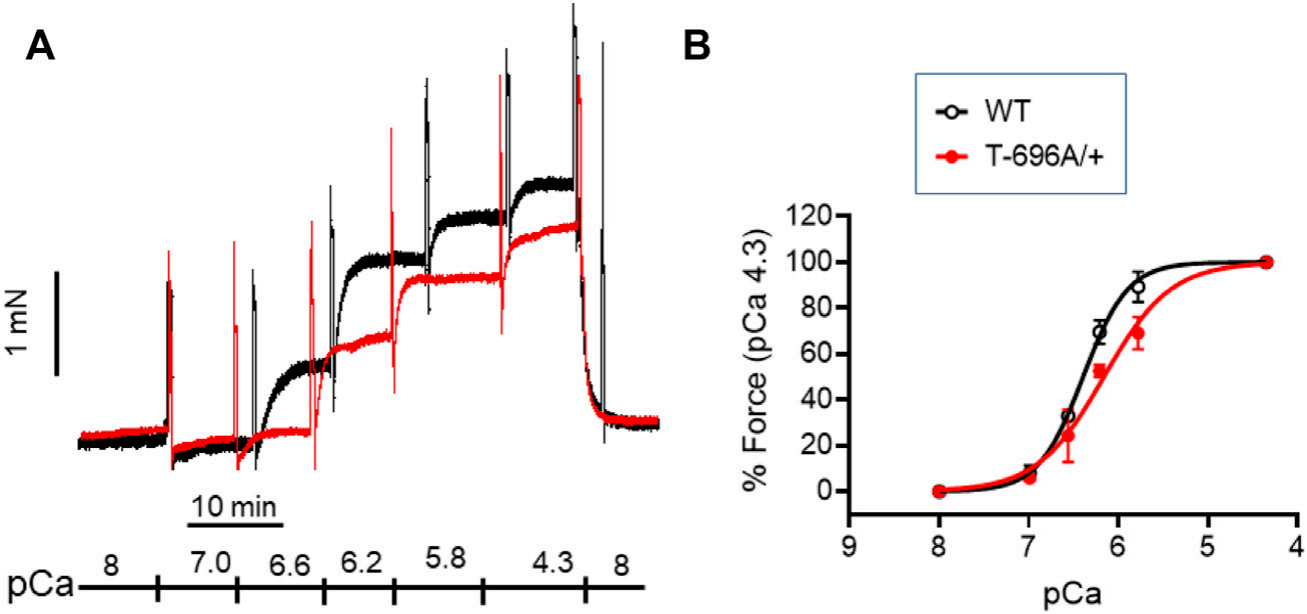
Ca^2+^-activated contraction of basilar arteries from young WT- and MYPT1-T696A/+-mice. Original force tracing **(A)** and statistical summary **(B)** depicting Ca^2+^-activated contraction of young BA from WT- and MYPT1-T696A/+-mice (*n* = 4–5). Tone normalized to pCa 4.3 accepted as 100%.

The level of phosphorylation of MYPT1-T696 was proposed to define the intrinsic Ca^2+^-sensitivity of the contractile machinery of urinary bladder smooth muscle (Khromov et al., 2009). To test whether this applies to basilar arteries, we obtained Ca^2+^-force relations in α-toxin permeabilized preparations. Permeabilization with α-toxin generates pores into the plasma membrane which allow diffusion of molecules <1 kDa into the intracellular space. This permits intracellular [Ca^2+^] to be controlled by EGTA so that the Ca^2+^-responsiveness of the contractile machinery can be measured without confounding changes in cytosolic [Ca^2+^]. After permeabilization, the preparations were equilibrated for 20 min in pCa >8 relaxing solution and then challenged with cumulatively increasing concentrations of [Ca^2+^] (Figure 3). We found that force in MYPT1-T696A/+ BA was lower than in WT BA at intermediate, but not at maximal [Ca^2+^] (Figure 3; Table 2). The pEC_50_, the neg. logarithm of concentration of Ca^2+^ for half maximal contraction, and the Hill coefficient of the Ca^2+^-force relation were lower than in WT (Table 2). Thus, under Ca^2+^-clamped conditions, the Ca^2+^-sensitivity was lower in mutant compared to WT vessels, consistent with a reduced inhibitory phosphorylation of MLCP. This result indicates that a lower Ca^2+^-sensitivity contributes to the lower L-NAME-induced tone in mutant y-BA.

**TABLE 2:** Ca^2+^-activated force in α-toxin permeabilized basilar arteries from WT and MYPT1-T696A/+ mice.

Vessel	pEC_50_ (pCa)	Hill slope (pCa)	F_max_ (mN)	n
WT	−6.4 ± 0.02	−1.8 ± 0.1	2.1 ± 0.6	4
T696A/+	−6.2 ± 0.04**	−1.2 ± 0.1*	1.9 ± 0.5^n.s.^	5

**pEC_50_ pCa curves WT vs. pCa curves T696A/+ (unpaired *t*-test).

*Hill Slope pCa curves WT vs. pCa curves T696A/+ (unpaired *t*-test).

^n.s.^F_max_ WT vs. T696A/+ (unpaired *t*-test).

### Expression of MYPT1-T696A/+ does not alter stretch-induced tone- and L-NAME-induced tone in senescent BA

**FIGURE 4:**
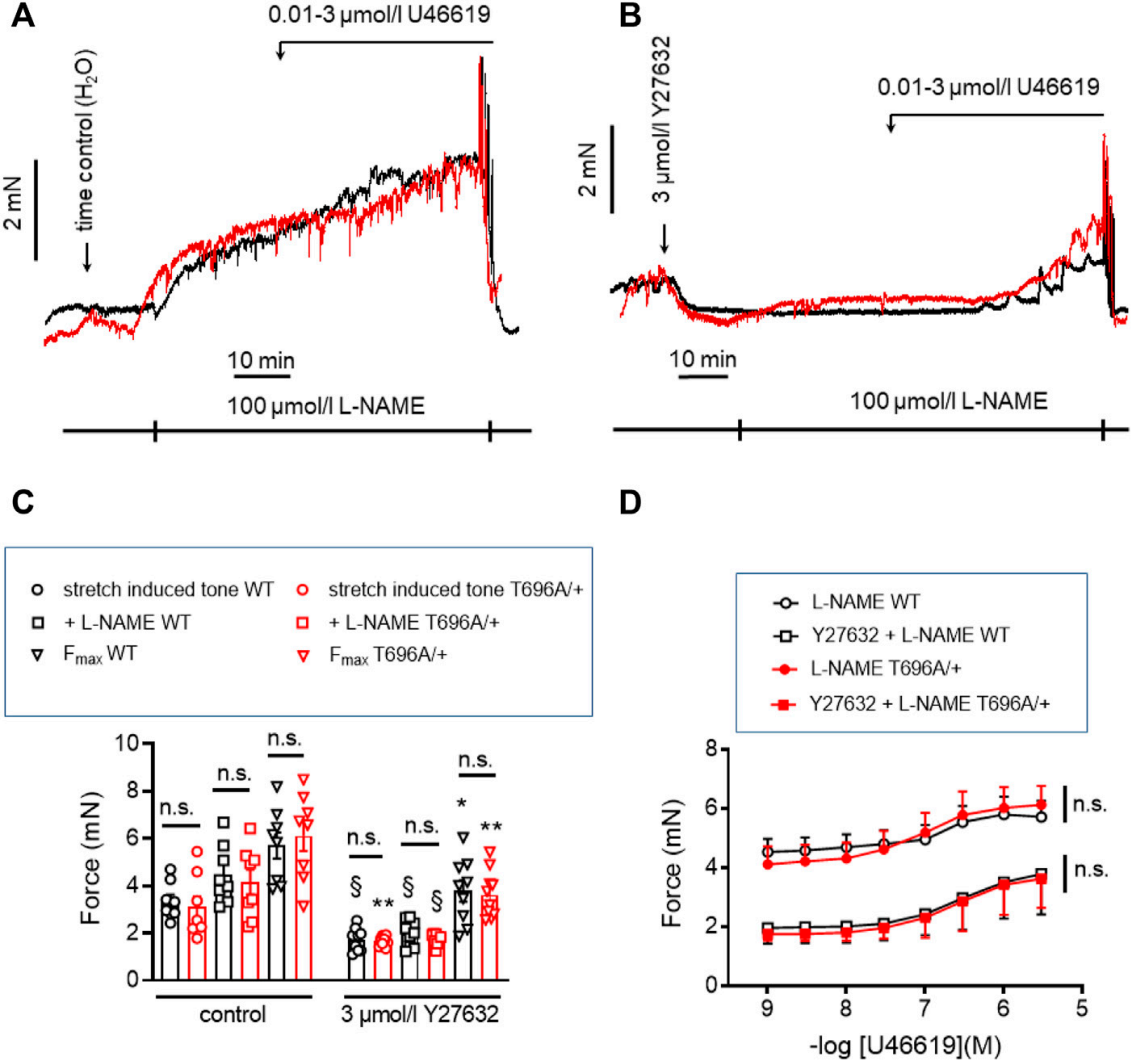
Stretch-, L-NAME-, and U46619-induced tone in arterial rings from senescent basilar arteries from WT and MYPT1-T696A/+ -mice under control conditions and under inhibition of ROK. **(A,B)** Original force tracings of the experiments performed with s-BAs from WT and MYPT1-T696A/+ -mice pretreated with vehicle [1% H_2_O; **(A)**] or 3 μmol/L Y27632 **(B)** and followed by inhibition of endogenous NO by treatment with 100 μmol/l L-NAME and cumulative application of U46619 (conc. 0.001–3 μmol/L). **(C)** Statistical evaluation of stretch-induced, L-NAME-induced and maximal tone (*n* = 8). **(D)** Statistical evaluation of tone induced by cumulative application of U46619 (*n* = 8). Data are represented as absolute force ±SEM; n.s. *p* > 0.05; two-way ANOVA. §*p* < 0.0001, stretch-induced tone WT controls (no treatment) vs. stretch-induced tone WT after 10 min treatment with 3 μmol/L Y27632, or stretch-induced tone L-NAME WT no treatment vs. stretch-induced tone L-NAME WT after 10 min treatment with 3 μmol/L Y27632, or stretch-induced tone L-NAME MYPT1-T696A/+ no treatment vs. stretch-induced tone L-NAME L-NAME MYPT1-T696A/+ after 10 min treatment with 3 μmol/L Y27632. *p < 0.05 F_max_ WT vs. F_max_ WT after treatment with Y27632; and **p < 0.001 stretch-induced tone MYPT1-T696A/+ controls (no treatment) vs. stretch-induced tone MYPT1-T696A/+ after 10 min treatment with 3 μmol/L Y27632 or F_max_ MYPT1-T696A/+ vs. F_max_ MYPT1-T696A/+ after treatment with Y27632. All values were calculated by unpaired *t*-test. n.s.—not significant; pEC_50_; ***p < 0.0001; two-way ANOVA.

In contrast to y-BA, in senescent BA (s-BA, >24 months old) a slow spontaneous rise in force was observed (Figure 4A) after stretching them to IC90 and equilibration in PSS for 20 min during the normalization procedure (see Methods). This tone amounted to 3.5 ± 0.9 mN in s-BAs from WT mice, which was not reduced in s-BA MYPT1-T696A/+ mice (3.1 ± 1.3 mN; Figures 4A, C; *n* = 8). The preparations from both genotypes were then incubated with 100 μmol/L L-NAME for 20 min, followed by stimulation with increasing concentrations of U46619 (0.001–3 μmol/L, Figure 4A). As in the experiments with y-BA, treatment of s-BA with 100 μM L-NAME increased tone in both mouse lines to a similar extent (4.5 ± 0.5 mN in WT vs. 4.2 ± 0.5 mN in mutant; *n* = 8). However, unlike the case with y-BA, the mutation did not blunt L-NAME induced tone (Figures 4A, C). U46619 concentration-response curves were similar in s-BAs from both mouse lines (Figures 4A, C, D; Table 3).

**TABLE 3:** Contractile parameters of intact preparations from senescent basilar arteries from wild type (WT) and heterozygous (MYPT1-T696A/+) mice.

Vessel	Stretch induced tone (mN)	L-NAME induced tone (mN)	pEC_50_ (U46619)	F_max_ (mN)	n
1) Controls (WT)	3.35 ± 0.3	4.5 ± 0.4	6.8 ± 0.1	5.7 ± 0,6	8
2) 3 µM Y27632 (WT)	1.74 ± 0.2^§^	1.98 ± 0.2^§, n.s.^	6.6 ± 0.1^§^	3.8 ± 0.5*	8
3) Controls (T696A/+)	3.10 ± 0.5^n.s.^	4.2 ± 0.6^n.s.^	6.9 ± 0.2	6.1 ± 0.7^n.s.^	8
4) 3 µM Y27632(T696A/+)	1.67 ± 0.1**^, n.s.^	1.75 ± 0.1^§, n.s.^	6.6 ± 0.1^n.s.,^^§^	3.6 ± 0.3**	8
5) Blebbistatin(+) (WT)	2.0 ± 0.3	3.1 ± 0.3	7.2 ± 0.1	5.7 ± 0.4^n.s.^	5
6) Blebbistatin(−) (WT)	1.95 ± 0.1*^, n.s^	2.01 ± 0.1^§, n.s.^	6.8 ± 0.03^n.s.,^ **	3.7 ± 0.2**	5
7) Blebbistatin(+) (T696A/+)	2.2 ± 0.3^n.s.^	3.0 ± 0.2 ^n.s.^	7.3 ± 0.2	6.0 ± 0.5 ^n.s.^	5
8) Blebbistatin(−) (T696A/+)	1.92 ± 0.2^§,n.s^	2.06 ± 0.2^§, n.s.^	6.9 ± 0.1^n.s.,^ **	3.6 ± 0.3**	5

Results: Stretch induced tone: ^§^ *p* < 0.0001 (1 vs. 2); **p < 0.01 (3 vs. 4 and 7 vs. 8); *p < 0.05 (5 vs. 6); n.s.—not significant (1 vs. 3 and 5 vs. 7); n.s.—not significant (2 vs. 4, 6, and 8); 2way ANOVA.

L-NAME induced tone: ^§^ *p* < 0.0001 (1 vs. 2, 3 vs. 4, 5 vs. 6 and 7 vs. 8); n.s.—not significant (1 vs. 3 and 5 vs. 7); n.s.—not significant (2 vs. 4, 6, and 8); 2way ANOVA.

pEC_50_ (U46619): n.s.—not significant (1 vs. 3, 1 vs. 5 and 1 vs. 7); ^§^ *p* < 0.0001 (1 vs. 2 and 3 vs. 4); **p < 0.01 (5 vs. 6 and 7 vs. 8); 2way ANOVA.

F_max_: n.s.—not significant (1 vs. 3, 1 vs. 5 and 1 vs. 7; 3 vs. 5 and 3 vs. 7; 5 vs. 7); *p < 0.05 (1 vs. 2); **p < 0.01 (3 vs. 4, 5 vs. 6 and 7 vs. 8); 2way ANOVA.

In y-BAs, inhibition of ROK blunted completely L-NAME-induced tone. We therefore investigated the effect of Y27632 in WT and MYPT1-T696A/+ s-BAs. U46619 concentration relationships and F_max_ after Y27632 treatment were similar between s-BAs from both groups (Figures 4B–D).

### Y27632 reduced phosphorylation of pMYPT1-T696/T853 and pMLC_20_-S19 in s-BAs

**FIGURE 5:**
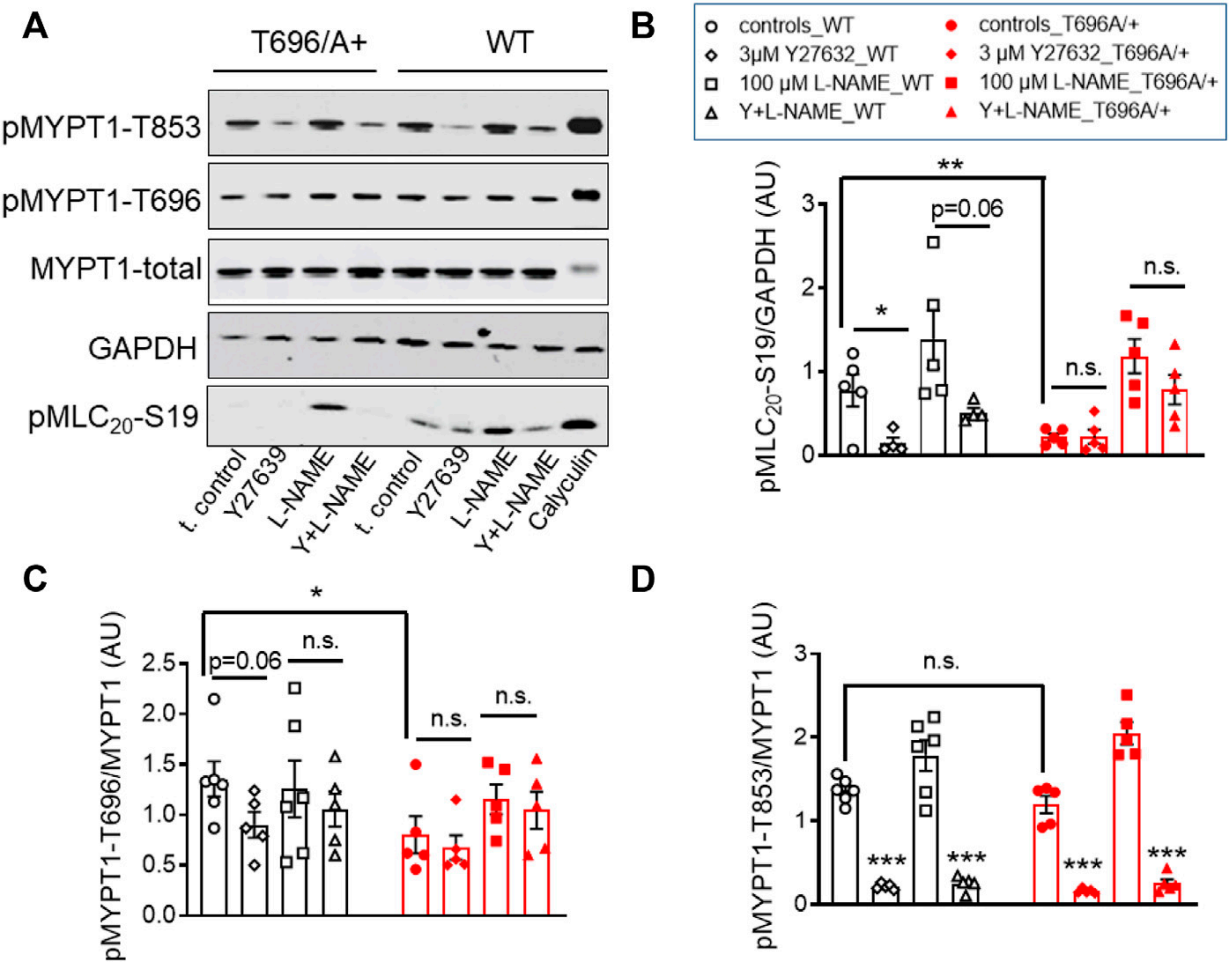
Effect of ROK inhibition on MLC_20_-S19, MYPT1-T696, and MYPT1-T853 in senescent basilar arteries from WT and MYPT1-T696A/+ mice. **(A)** Original western blots and statistical evaluation of the phosphorylation of **(B)** MLC_20_-S19 (pMLC_20_), and MYPT1 at **(C)** T696 **(**pMYPT1-T696) and **(D)** T853 (pMYPT1-T853) in s-BAs from WT and MYPT1-T696A/+ mice under control conditions (no stimulations), and after preincubation with 3 μmol/L Y27632, 100 μmol/l L-NAME or Y27632 plus L-NAME. Data are represented as ratio of pMYPT1-T696/853 toward MYPT1-total or MLC_20_-S19 toward GAPDH (*n* = 6). **p < 0.01, *p < 0.05, n.s.—not significant; two-way ANOVA. n.s.—not significant; ***p < 0.0001 and *p* = 0.06 have been calculated by using unpaired *t*-test.

In s-BAs from MYPT1-T696A/+ mice, the basal pMLC_20_-S19 and pMYPT1-T696 were lower than in WT arteries (Figures 5A–C). Incubation with 3 μmol/L of Y27632 reduced pMLC_20_-S19 only in WT s-BAs (Figures 5A, B). pMYPT1-T853 in WT and mutant s-BAs did not differ under basal conditions or L-NAME-, or Y27631-treatment (Figures 5A, D).

### Non-muscle myosin II is involved in stretch-induced tone and L-NAME-induced tone

**FIGURE 6:**
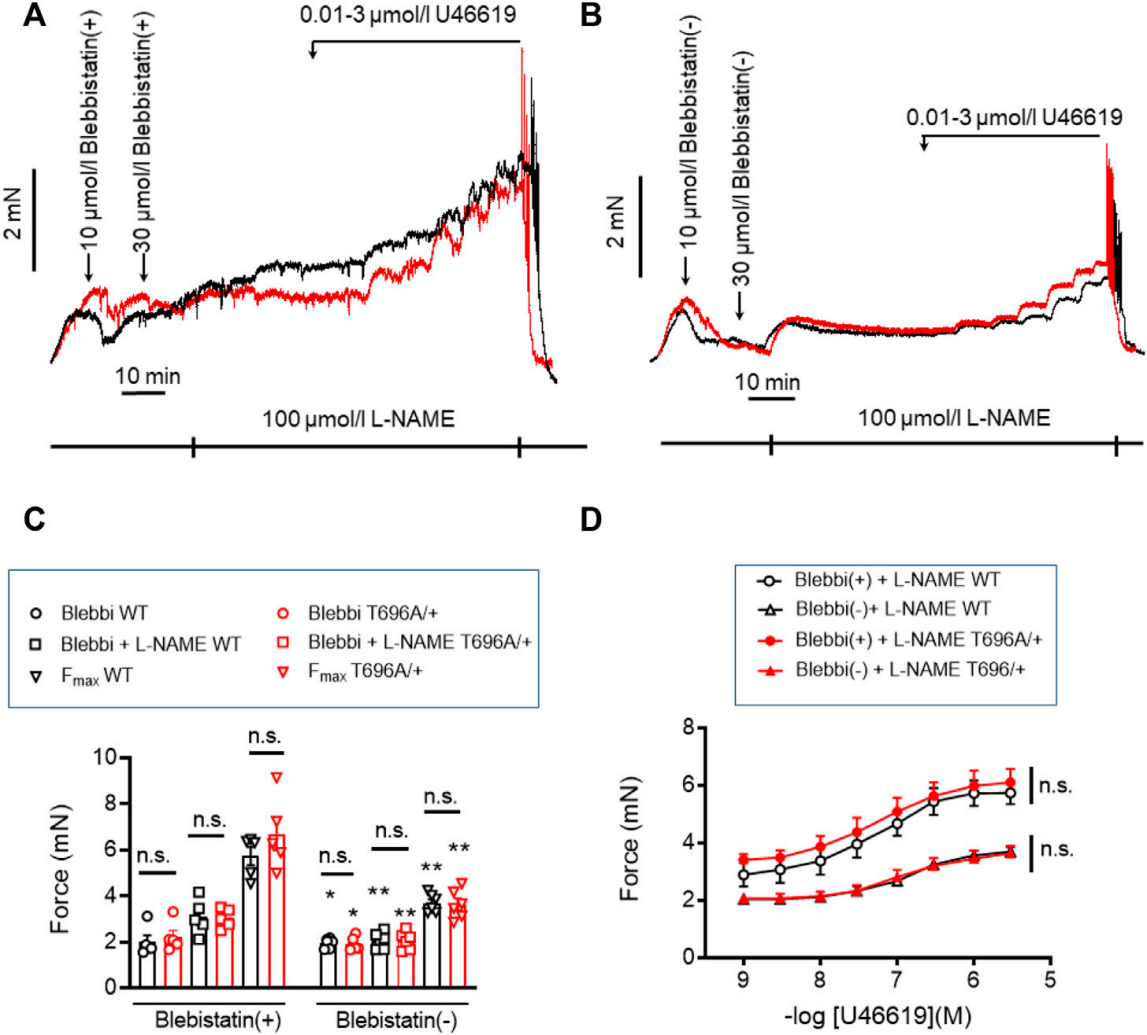
Stretch-, L-NAME-, and U46619-induced tone in senescent basilar arteries from WT and MYPT1-T696A/+ -mice under inhibition of cross-bridge cycling of non-muscle myosin. **(A,B)** Original force tracings representing the effect of the inhibitor of cross-bridge cycling of non-muscle myosin, blebbistatin on tone. Vessels were pretreated with the active (−) and inactive (+) enantiomers of blebbistatin and further incubated with 100 μmol/l L-NAME and cumulative application of U46619. **(C)** Statistic evaluation of stretch-induced, L-NAME-induced and maximal tone in presence of (+) or (−) blebbistatin (*n* = 5). **(D)** Statistic evaluation of tone induced by cumulative application of U46619 (*n* = 5). Data represented as absolute force ±SEM; n.s. *p* > 0.05; two-way ANOVA. *p < 0.05 and **p < 0.001 calculated by unpaired *t*-test.

Stretch-induced tone and L-NAME induced tone development under resting conditions, i.e., in PSS, is much slower than agonist induced contraction. Such slow force development has been observed in neonatal mice, in which smooth muscle myosin II was genetically ablated and force development was ascribed to non-muscle myosin (NM-II) (Morano et al., 2000). Blebbistatin has been used as a small molecule inhibitor of NM-II (Limouze et al., 2004; Rhee et al., 2006), but its specificity has recently been questioned as it was reported to also inhibit smooth muscle myosin II (Eddinger et al., 2007). The active (−) form of blebbistatin reduced stretch-induced tone and L-NAME induced tone in s-BAs to a similar degree as Y27632 (Figures 6A–C; Table 3). Under (+) blebbistatin treatment stretch-induced tone was 2.0 ± 0.3 mN vs. 2.2 ± 0.3 mN in heterozygous s-BAs from MYPT1-T696A/+ animals and amounted to 3.1 ± 0.3 mN and 3.0 ± 0.2 mN after 20 min treatment with L-NAME (*n* = 5; Figure 6A; and Table 3). In 2 of 5 experiments application of 30 µmol/L (+) Blebbistatin had no additional effect on tone. The concentration responsiveness and F_max_ of U46619 in the presence of blebbistatin (−) or (+) were similar to those measured previously in Y27632 treated group (Figures 6A, C, D; Table 3).

### Blebbistatin (−) unlike Y27632 did not reduce phosphorylation of pMYPT1-T696/T853 and pMLC_20_-S19 in s-BAs

**FIGURE 7:**
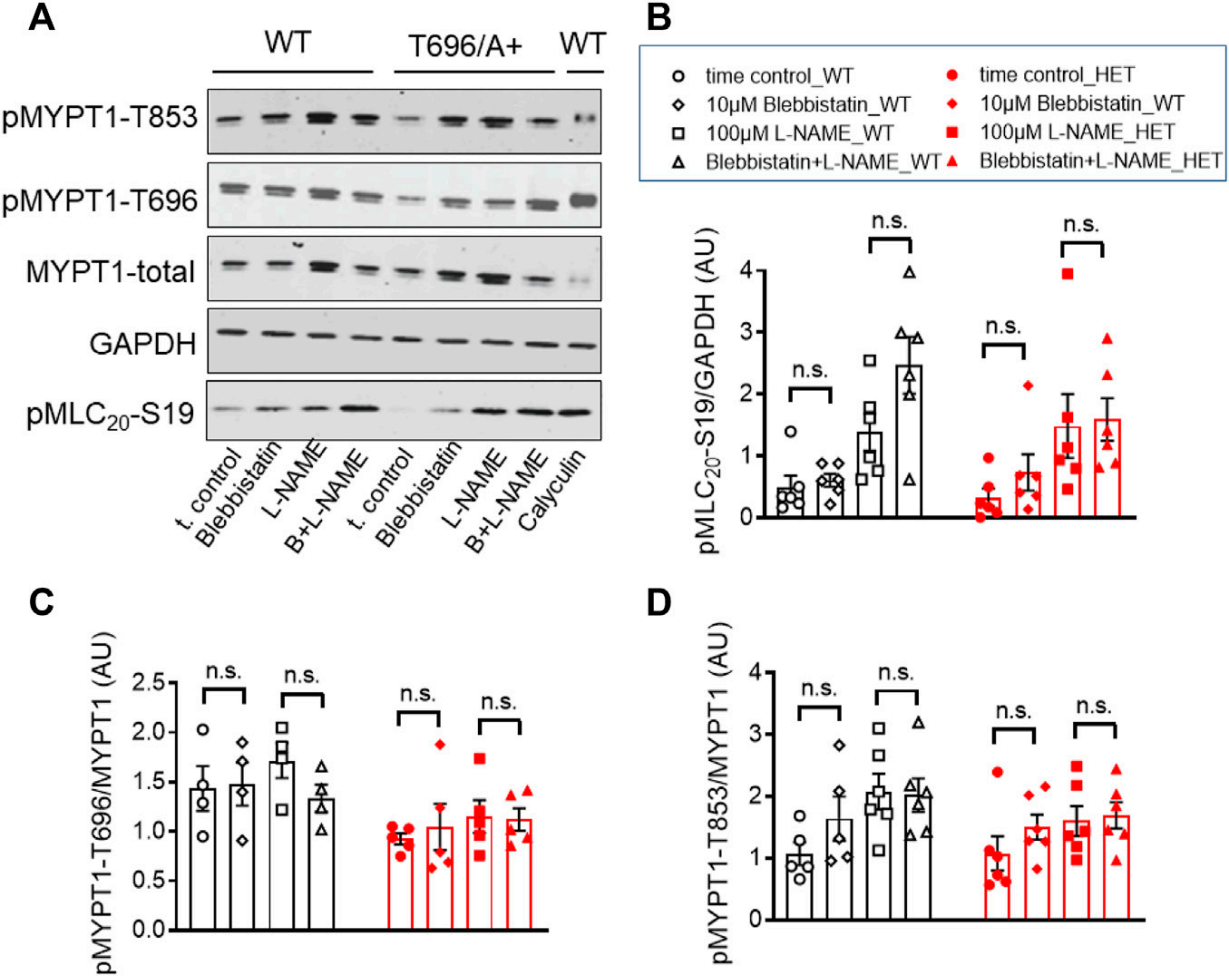
Phosphorylation of pMYPT1-T696, pMYPT1-T853, and pMLC_20_-S19 in senescent basilar arteries from WT and MYPT1-T696A/+ mice under inhibition of cross-bridge cycling of non-muscle myosin. **(A–D)** Original Western blots **(A)** and statistic evaluation of the phosphorylation of pMLC_20_-S19 **(B)**, pMYPT1-T696 **(C)** and pMYPT1-T853 **(D)** in s-BAs from WT and MYPT1-T696A/+ mice under control conditions and after inhibition of cross-bridge cycling of non-muscle myosin by blebbistatin (−). Preparations mounted as previously and treated by vehicle (0.3% DMSO; controls), or 10 μmol/L blebbistatin (−), or 100 μmol/l L-NAME, or blebbistatin (−) plus L-NAME. Data represented as ratio of pMYPT1-T696/853 toward MYPT1-total or MLC_20_-S19 toward GAPDH (*n* = 5). n.s.—not significant; unpaired *t-test.*

Next we tested whether blebbistatin reduced basal MLC_20_-S19 phosphorylation or inhibitory phosphorylation of MYPT1. Phosphorylation of pMLC_20_-S19, pMYPT1-T696, and pMYPT1-T853 in the presence of blebbistatin (−) was determined in s-BAs from WT and from MYPT1-T696A/+ mice using phosphospecific antibodies. Neither pMLC_20_-S19, nor pMYPT1-T696/T853, were reduced by blebbistatin (−) in BAs from both mouse lines (Figures 7A–D). These experiments support the notion that the hyper-contractile phenotype of senescent BAs involves increase in ROK activity and likely non-muscle myosin-II cross-bridge cycling.

### Phosphorylation of non-muscle myosin-II in young and senescent BAs

**FIGURE 8:**
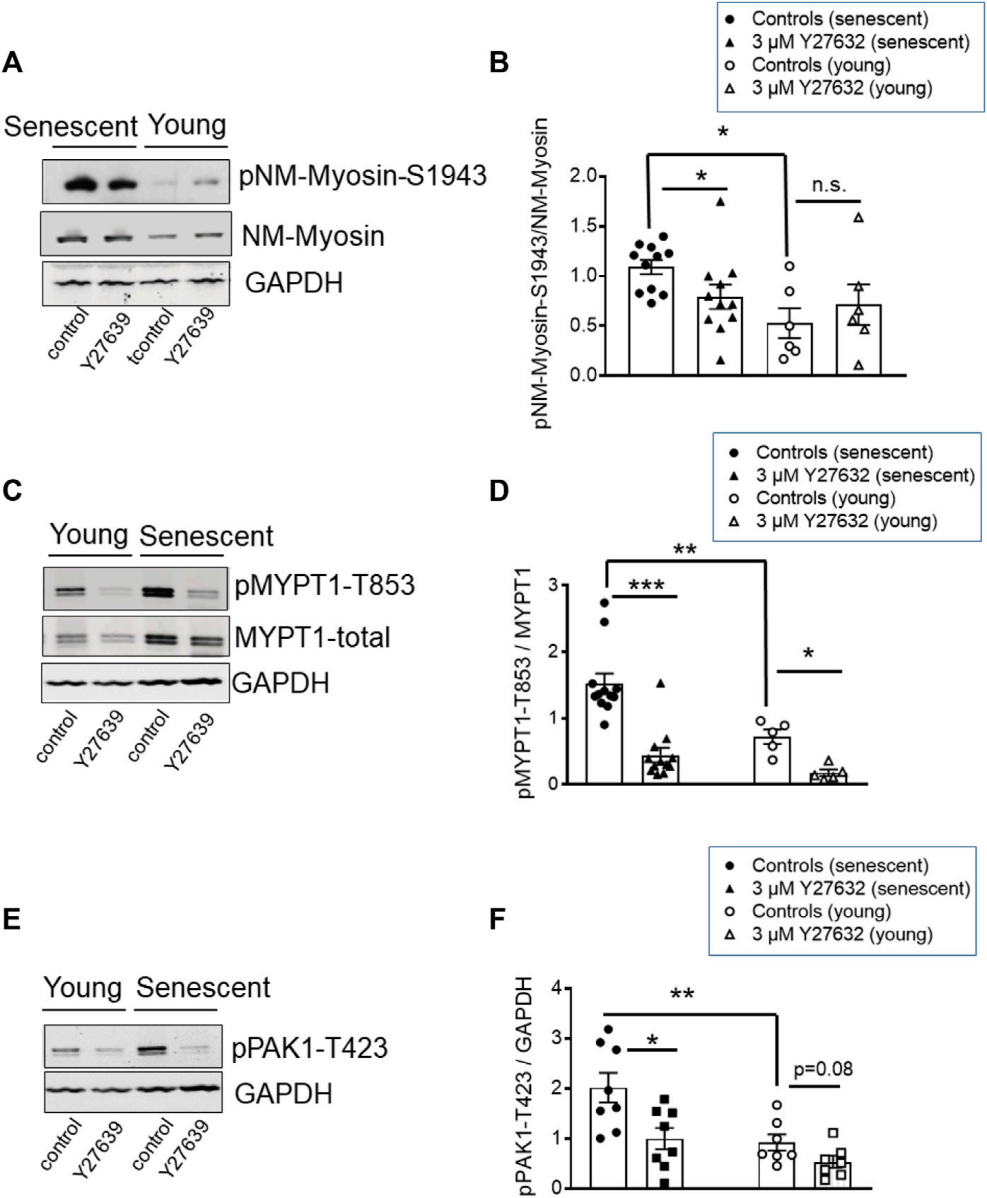
Phosphorylation of NM-II-S1943, MYPT1-T853, and PAK-T423 in basilar arteries from young and senescent mice under control conditions and after inhibition of ROK. **(A–C)** Original western blots and statistic evaluation of the phosphorylation of MYPT1 at T853 **(A,B)**, non-muscle-myosin II (NM-Myosin) at S1943 **(C,D)** and PAK-T423 **(E,F)** in y- and s-BAs from WT animals (*n* = 10–6). Preparations were treated either by vehicle (0.5% H_2_O; controls) or by 3 μmol/L Y27632, shock-frozen and subjected to Western blot as described in “Methods.” **p < 0.01 and *p < 0.05; control y-BAs vs. control s-BAs; two-way ANOVA. **p < 0.001 and *p < 0.05; unpaired *t*-test.

Contractile activity requires that NM myosin-II proteins assemble into filaments. A predictor of filament competent NM myosin is the level of phosphorylation of its heavy chains (MHC) at S1943 (Zhang and Gunst, 2017). Here, we tested 1) whether NM-II S1943 phosphorylation was higher in senescent than in young arteries, 2) whether Y27632 decreased phosphorylation of this site and 3) whether the expression pattern of global or IIa and IIb isoforms of NM-II are altered by senescence. Phosphorylation of NM-MHC-S1943 was quantified by Western blot analysis using phosphospecific antibodies. In s-BAs, basal S1943 phosphorylation was significantly higher under control conditions (time matched controls to the Y-27632 treated preparations) than in y-BAs (Figures 8A, B). Incubation with 3 μmol/L Y27632 reduced the immunoreactivity against pNM-II-S1943 in s-BAs, but not in y-BAs (Figures 8A, B). No difference in the expression levels of NM-II-total between y- and s-BAs was observed (Supplementary Figures S5A, B). Interestingly, using the same scan intensity, the intensity of the immunoreactive signal obtained with NM-IIa antibodies was larger than with NM-IIb antibodies, supporting the view that NM-IIa is the predominant isoform in BAs of both ages (Supplementary Figures S5A, C, D).

In these experiments, phosphorylation of MYPT1-T853 as a marker for ROK-activity was monitored in parallel. Phosphorylation of T853 was significantly higher in s-BAs than in y-BAs. In both age groups, Y27632 strongly reduced the immunoreactivity of pMYPT1-T853 (Figures 8 C, D). These experiments indicate that ROK regulates NM-II S1943 phosphorylation.

### Senescence increased basal phosphorylation of PAK1-T423, a down-stream effector of ROK

PAK1 ist another target of ROK, which phosphorylates PAK1 at threonine 432 and thereby activates the enzyme (Zhang et al., 2018). We assessed the phosphorylation level of PAK1 using phosphospecific antibodies against pPAK1-T432 in Western Blots prepared with the same samples we used for analysis of NM-II-S1943- and MYPT1-T853-phosphorylation. The immunoreactive signal of PAK1-T423 in s-BAs was higher than in y-BAs. In both groups, incubation with Y27632 reduced the PAK1-T423 phospho-signal (Figures 8E, F).

### Early onset of stretch-induced tone in Cald1^+/−^ old basilar arteries (o-BAs)

**FIGURE 9:**
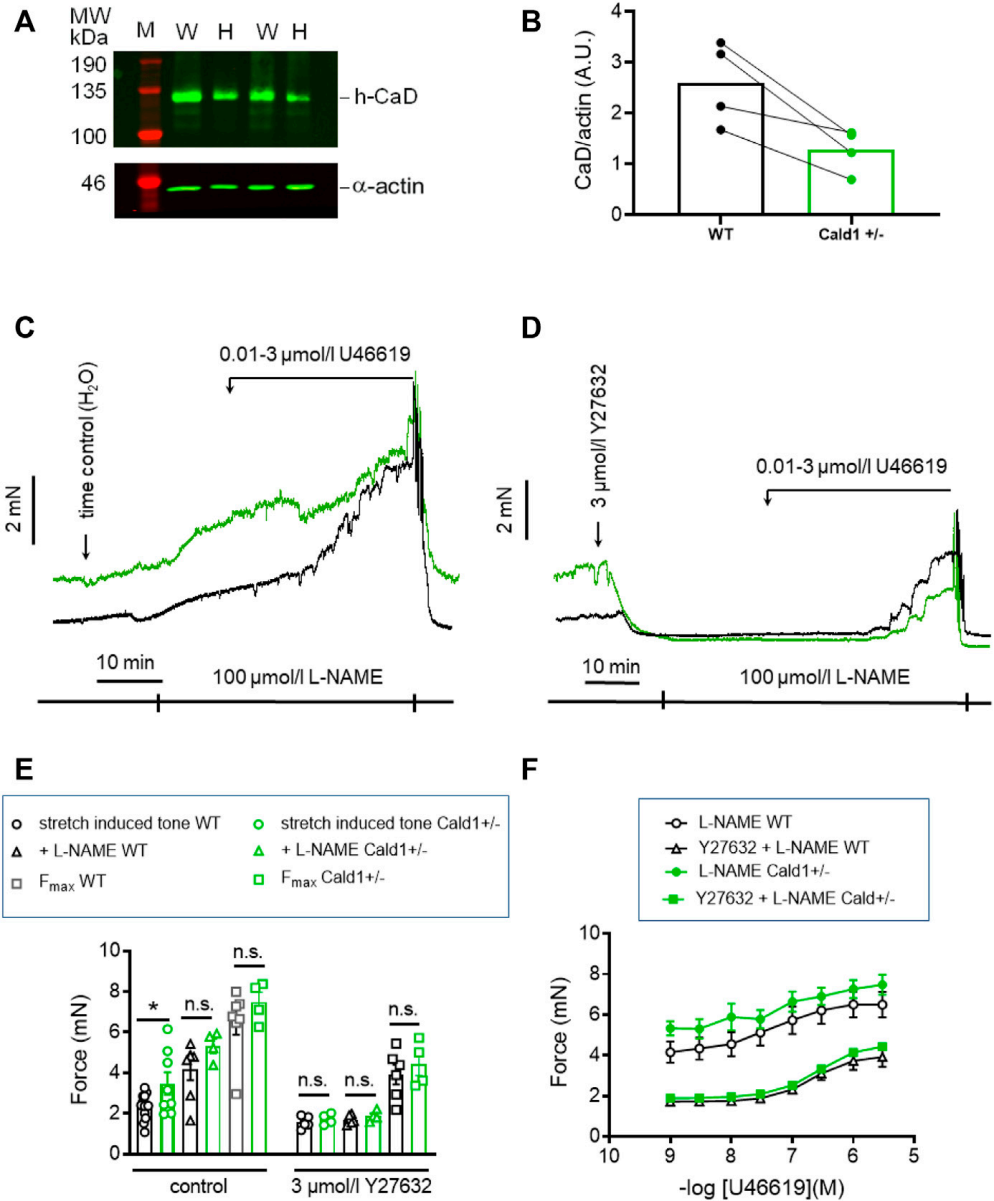
Effect of Caldesmon targeting on tone maintenance in basilar arteries from old mice. **(A)** Representative Western blot of expression of h-CaD in WT and heterozygous s-BAs from 2 litters **(B)** statistical evaluation from arteries of four independent measurements (*n* = 3 litters with WT and Het littermates, *n* = 1 WT and Het from different litters). M, marker; W, wilde type; H, heterozygotes. Caldesmon expression the Ponceau Red stained actin band. **(B)** Statistic evaluation. **(C,D)** Original force tracings representing the effect of caldesmon mutation on tone. **(E)** Statistic evaluation of stretch-induced, L-NAME-induced and maximal tone in BAs from WT and Cald1^+/−^ BAs (*n* = 6–4). **(F)** Statistic evaluation of tone induced by cumulative application of U46619 (*n* = 6–4). Data represented as absolute force ±SEM; *p < 0.05; n.s. *p* > 0.05; two-way ANOVA.

PAK1 has several downstream targets involved in regulation of tone. One of them, caldesmon was reported to elicit a contraction without an increase in MLC_20_ phosphorylation when phosphorylated by PAK1 (Van Eyk et al., 1998). Thus, phosphorylation of caldesmon by PAK1 and reversal of its inhibitory effect on cross-bridge cycling is a potential mechanism that could account for the stretch-induced tone in s-BAs. Unfortunately, there are no commercial phosphospecific antibodies available to test whether caldesmon was phosphorylated by PAK1. Therefore, we investigated the involvement of caldesmon in the regulation of stretch-induced tone in arteries from heterozygous mice, carrying one allele in which Cald1 gene was deleted by homologous recombination of the *Cald1* gene (Putz et al., 2021). As homozygosity is prenatally lethal, only heterozygous mice (Cald1^+/−^) were employed (Putz et al., 2021). In heterozygous old basilar arteries (20–21 months old; o-BAs) expression of caldesmon was ∼50% lower than in WT mice (*n* = 4, Figures 9A, B). Interestingly, stretch-induced tone was observed in o-BAs obtained from heterozygotes Cald1^+/−^ mice at an earlier age of 20–21 months, compared to the BAs from controls MYPT1-T696A/+-mice in which such a tone appeared at senescence (age >24 months). At this age, o-BAs from WT mice did not exhibit stretch-induced tone yet (Figures 9C, D). The magnitude of stretch-induced tone of o-BAs from Cald1^+/−^ mice was similar to that in WT s-BAs (3.5 ± 0.9 mN in s-BAs from WTs vs. 3.5 ± 0.6 mN in o-BAs from Cald1^+/−^; *n* = 8). There was no difference between both genotypes considering L-NAME- and U46619-induced tone (Figures 9C, E). As in previous experiments, application of 3 μmol/L Y27632 reduced stretch-induced tone in both groups (Figures 9C–F). We did not observe significant differences in stretch-induced or L-NAME-induced tone, nor in maximal -induced tone between y-BAs from WT and Cald1^+/−^ mice (12–14 weeks old) (Supplementary Figure S4).

**FIGURE 10:**
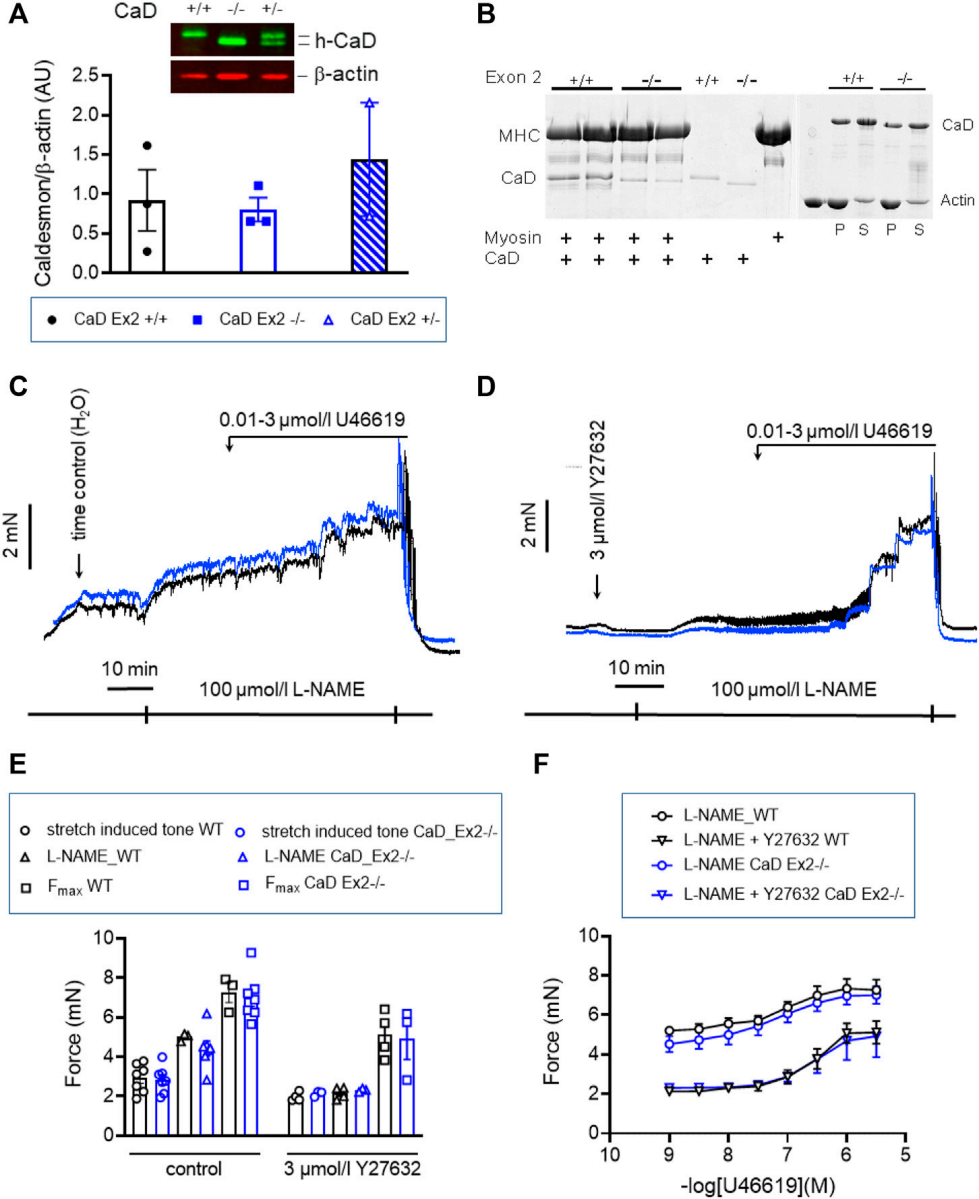
Effect of ablation of exon2 (Ex2) of Caldesmon on tone maintenance basilar arteries **(A)** Original luminogram and statistic evaluation showing caldesmon expression in s-BAs obtained from wild type animals, homozygous animals lacking the strong myosin-binding domain of caldesmon encoded by Exon 2 (−/−) and heterozygous animals (+/−) (*n* = 3-3-2). **(B)** Analysis of two independent experiments confirming the lack of myosin binding of the truncated caldesmon isolated from Cald1 −/− mice (left panel), whereas actin-binding was not affected (right panel). **(C,D)** Original force tracings representing the effect of ablation of Exon2 in caldesmon protein on tone. **(E)** Left: Statistic evaluation of stretch-induced (*n* = 7), L-NAME-induced (*n* = 3–7), and maximal tone (*n* = 3–7) in BAs from WT (CaD_ΔEx2 +/+) and CaD ΔEx2^−/−^ BAs under control conditions. Right: Statistic evaluation of stretch-induced (*n* = 4–3), L-NAME-induced (*n* = 4–3), and maximal tone (*n* = 4–3) in BAs from same groups in the presence of 3 μmol/L Y27632. **(F)** Statistic evaluation of tone induced by cumulative application of U46619 (*n* = 6–4). Data represented as absolute force ±SEM.

Inhibition of cross-bridge cycling by caldesmon is exerted by the C-terminal, actin-binding domain (Pfitzer et al., 1993b). Binding to myosin was proposed to position caldesmon in such a way that the actin-binding domain can inhibit cross-bridge cycling (Lee et al., 2000). We tested this in a second mouse line, CaD-ΔEx2^−/−^, in which expression of the strong myosin binding domain, which is encoded by Exon 2 of the Cald1 gene, was ablated (Figure 10) (Pfitzer et al., 2005). Expression of the truncated CaD did not differ from WT (Figure 10A). Whereas WT CaD bound to myosin in solution, binding was nearly abolished in truncated caldesmon (CaD-ΔEx2^−/−^) (Figure 10B). Binding to actin was not affected (Figure 10B). o-BAs from homozygous mice expressing the truncated caldesmon were subjected to the same protocols used in previous experiments. No significant change of vascular tone in o-BAs from CaD-ΔEx2^−/−^ mice compared to their time matched WT controls was observed (Figures 10C–F).

### Basal G/F-actin ratio and localization of F-actin in senescent BAs after maximal stimulation

**FIGURE 11:**
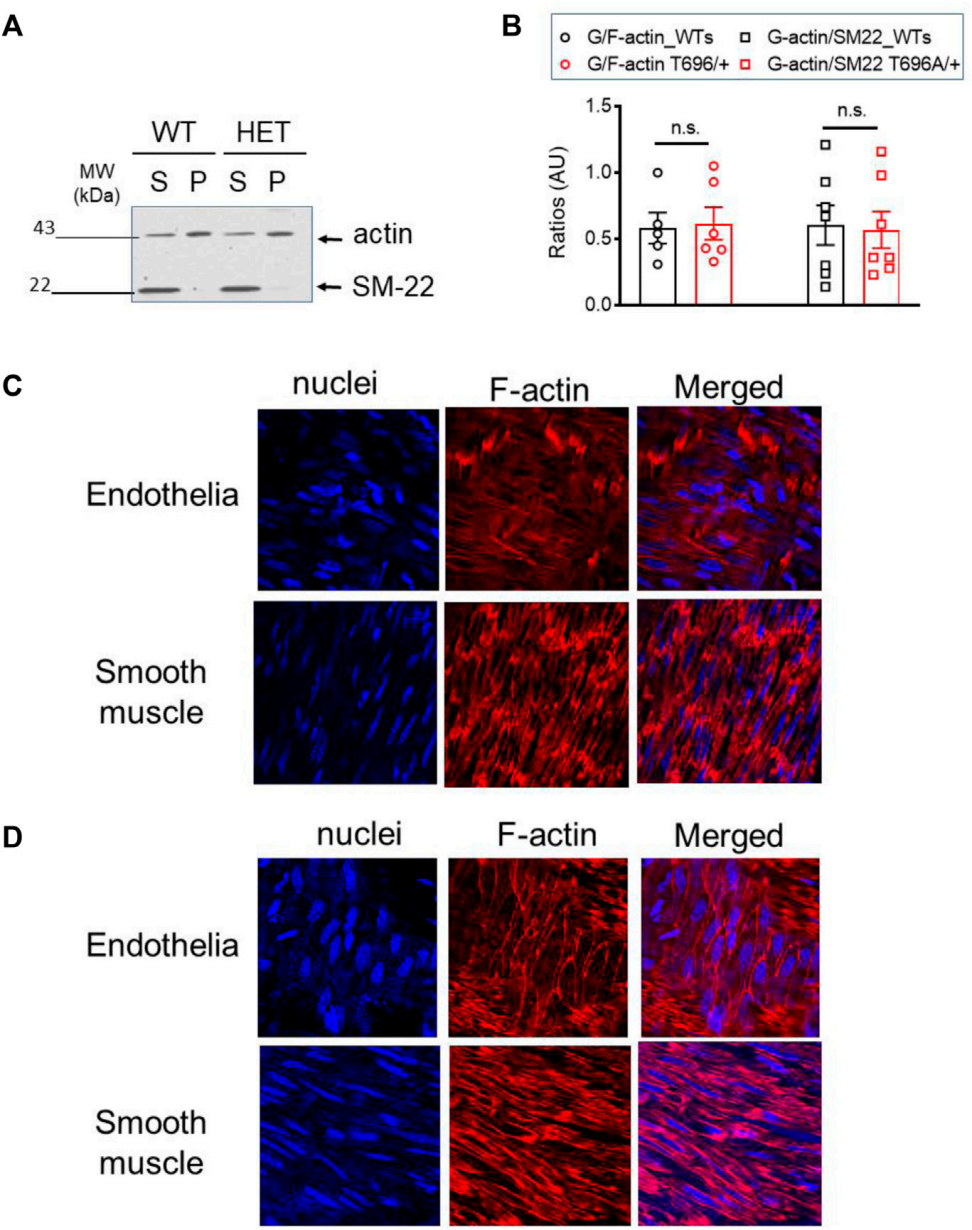
G/F actin ratio in basilar arteries from senescent WT and MYPT1-T696A/+ animals under basal conditions. Localization of F-actin in basilar arteries from senescent WT mice after Calyculin stimulation **(A)** Original Western blot representing the global actin-immunoreactivity in supernatant (S) used as read out for globular (G) -actin fraction and pellet (P) used as read out for fibrillar (F) -actin in s-BAs from WT and MYPT1-T696A/+ animals. Supernatant and pellet fractions were obtained by ultracentrifugation as described in methods. **(B)** Statistic evaluation of *n* = 6 s-BAs, each group. n.s.—not significant; *p* > 0.05; two-way ANOVA. Confocal images y-BAs **(C)** and s-BAs **(D)** arteries from WT animals stained with phalloidin for F-actin and Hoechst for Nuclei. Transmission light images are denoted as “T-light.” Vessels were isolated, mounted and normalized as described in methods and treated for 30 min with 0.1 μmol/L Calyculin. After stimulation, preparations were fixed and F-actin was stained with Fluor™ 555 conjugated phalloidin, nuclei were stained with Hoechst 33342 (see methods). Representative images from four independent vessels/animals.

The G/F-actin decreased in a ROK-dependent manner by agonist stimulation of airway smooth muscle cells (Zhang and Gunst, 2017) or under basal conditions in old BAs (Lubomirov et al., 2017). To validate that targeting of MYPT1-T696 does not influence F-actin polymerization, we next measured the G/F-actin ratio in s-BAs from WT and MYPT1-T696A/+ animals. No change of G-actin immunoreactivity in supernatants, used as read out for the G-actin fractions, was detected in BAs from WT compared to MYPT1-T696A/+ animals (Figures 11A, B).

In the next series of experiments, we investigated whether maximal stimulation with calyculin resulted in a redistribution of F-actin in favor of subcortical actin. Calyculin treatment induced a maximal increase in force, starting 7–10 min after application and reached its maximum after 30 min (not shown). In the previous experiments, we showed that in BAs, calyculin treatment maximally activates ROK as seen by phosphorylation of MYPT1-T853 (Figure 7A). Alexa Fluor™ 555 phalloidin staining of F-actin filaments showed changes in localization in endothelial and smooth muscle cells. In s-BA endothelial cells F-actin localized to the cortex, while F-actin, stained by Alexa Fluor™ 555 phalloidin, in endothelium of y-BAs was diffuse and could not be attributed to a subcellular niche (Figures 11C, D). Interestingly, in smooth muscle cells from y-BAs F-actin localized predominantly to the cell-poles (Figure 11C).

## Discussion

Stretch-induced vascular tone is an important element of autoregulatory adaptation of the cerebral circulatory system. It maintains constant cerebral flow, despite changes in perfusion pressure, and thus ensures adequate brain perfusion. The present work provides novel insights into the mechanisms of regulation of the stretch-induced tone of murine BA in senescence. 1) In young BAs (y-BAs) mechanical stretch did not lead to spontaneous tone generation, while the senescent-BAs (s-BAs) developed stable stretch-induced tone. 2) Stretch-induced tone in y-BAs appeared only after inhibition of NO-release by L-NAME, whereas in s-BAs such an inhibition led to an additive effect. 3) In BAs from both age groups, stretch-induced tone was fully inhibited by treatment with a ROK-inhibitor, Y27632. 4) Lowering of the phosphorylation propensity of MYPT1 in heterozygous mice carrying the point mutation MYPT1‐T696A/+ prevented stretch-induced tone in y-BAs but not in aged BAs. Basal MLC_20_ phosphorylation was lower in MYTP1-T696A/+ than in WT BAs in both age groups. Based on this, we propose that regulation of stretch-induced tone in senescent arteries involves alternative mechanism. 5) The basal phosphorylation level of NM-II and PAK1, which phosphorylates and inhibits caldesmon, were higher in s-BAs than in y-BAs, and were reduced by the ROK inhibitor, Y27632. 6) Ablation of caldesmon augmented stretch-induced tone in old BAs suggesting that it acts as a molecular brake on stretch-induced tone in aged BAs. We propose that in senescent cerebral vessels, mechanisms distinct from MLC_20_ phosphorylation contribute to regulation of tone in the absence of a contractile agonist.

### The onset of senescence is associated with MLC_20_-independent mechanisms of cerebrovascular tone regulation

There is ample evidence that stretch-induced cerebrovascular tone in young arteries relates to two important components, namely, 1) the plasma membrane depolarization, leading to a rise in intracellular Ca^2+^-concentration and activation of the specific Ca^2+^/calmodulin-dependent protein kinase, MLCK, and 2) Ca^2+^-sensitization *via* inhibition of MLCP (Walsh and Cole, 2013), both inducing an increase in phosphorylation of MLC_20_. Here, we show that there is a marked difference in the mechanical behavior of y-BAs and s-BAs and its regulation in the absence of a contractile agonist. In both y-BAs and s-BAs, there is a rapid rise in tone followed by a decline to a lower level upon stepwise stretching the vessels to IC90 during the normalization procedure as expected from the length tension relation. The tone at IC90 is stable in y-BAs during the whole experiment, i.e., there is no slow increase in tone, and basal tone recovers after washout of the contractile agonist U46619. In contrast, in s-BAs tone at IC90 is not stable, but rather slowly increases. This rise in tone we denote as stretch-induced tone. In y-BAs, such a stretch-induced tone was observed only after inhibition of NO-signaling by L-NAME.

At both ages, the developed stretch-induced tone and L-NAME induced tone were inhibited by the ROK inhibitor Y27632, putting ROK center stage in regulating tone in basilar arteries, noteworthy, even in the absence of a contractile agonist. Our experiments indicate that not only NO-PKG signaling (Lubomirov et al., 2017; Lubomirov et al., 2018), but also ROK signaling is constitutively active in BAs. In y-BAs, NO-PKG signaling, known to inhibit RhoA-ROK-MLCP signaling at different levels along the pathway, prevents the spontaneous rise in force, while in s-BAs, the balance between NO-PKG and ROK signaling is shifted in favor of the latter. The mechanisms that underlie the constitutive activity of ROK unmasked by inhibition of NO-release are currently unknown. It is possible that stretching the vessels, either by opening of stretch sensitive cation channels (Mederos y Schnitzler et al., 2011) and/or by activating G-protein receptors (Momotani et al., 2011; Takefuji et al., 2013) leads to a Ca^2+^-influx, which then *via* tyrosine kinase Pyk2 might activate Rho-ROK signaling [(Mills et al., 2015); reviewed in (Brozovich et al., 2016)]. It is of interest that in aged renal vessels phosphorylation and activity of 90 kDa ribosomal S6 kinase (RSK2) are higher than in young renal vessels (Lubomirov et al., in press). RSK2 is a non-canonical MLC_20_-kinase (Suizu et al., 2000; Artamonov et al., 2018) and also activates ROK (Shi et al., 2018), and hence may be responsible for stretch-induced tone in s-BAs. We are currently exploring this possibility.

The downstream targets of ROK appear to differ between y-BA and s-BA. In y-BA, ROK appears to act through the canonical pathway, i.e., ROK-induced inhibitory phosphorylation of MYPT1 at T696 and T853, resulted in an increase in phosphorylation of MLC_20_ [reviewed in Puetz et al. (2009)]. We do not exclude the possibility that a rise in intracellular Ca^2+^-concentration also leads to MLC_20_ phosphorylation and increase in tone. The ROK inhibitor, Y27632, lowers phosphorylation of both sites, thus increasing the activity of MLCP, which in turn lowers MLC_20_ phosphorylation. Y27632 prevents the increase in L-NAME-induced force. However, which one of the two residues or perhaps both are responsible for MLCP inhibition is under debate (Feng et al., 1999; Khromov et al., 2009). To address this, we used a mouse model (MYPT1-T696A/+ mice), in which the phosphorylation propensity of T696 was genetically lowered. In y-BAs, the mutation prevented L-NAME induced tension and phosphorylation of MLC_20_, as did Y27632.

Surprisingly, stretch-induced tone in s-BAs, although ROK dependent, appears not to be mediated by MLC_20_ phosphorylation. This notion is based on the observation that basal MLC_20_ phosphorylation but not stretch-induced tone was reduced in s-BAs carrying the MYPT1-T696A/+ mutation. In other words, stretch-induced tone develops in s-BAs despite low levels of MLC_20_ phosphorylation. As the vessels for phosphorylation determination were not stretched because of technical reasons, albeit pharmacological treatment was identical to the mechanical experiment, we cannot exclude the possibility that stretching the vessel would have increased MLC_20_-phosphorylation. These data are in contrast to our previous observation, where the T696A mutation reduced both, phosphorylation of MLC_20_ and tone in young and old cerebral arteries (Lubomirov et al., 2017; Lubomirov et al., 2018). It appears from our study that regulation of cerebrovascular tone in young and old age occurs predominantly through MLC_20_ phosphorylation, the canonical pathway, whereas in senescence these pathways play a minor role.

### NM-II and PAK1, important ROK downstream-effector-proteins, regulate stretch-induced tone in senescence

Several non-canonical pathways, in addition to the canonical one, have been shown to regulate tone in tracheal (Zhang and Gunst, 2019), and vascular smooth muscle (Kim et al., 2010; Moreno-Dominguez et al., 2013) by acting through both actin and NM-II. A series of recent publications demonstrated that ROK regulates polymerization of subcortical actin at cell adhesomes in response to contractile stimuli (Zhang et al., 2016; Zhang et al., 2018). Actin polymerization and assembly are multistep processes involving a large number of other regulatory proteins.

Furthermore, according to Gunst and co-workers (Zhang et al., 2016; Zhang et al., 2018), key events are RhoA mediated NM-II filament assembly, as well as ROK mediated phosphorylation and activation of PAK1. Our study suggests that these events are also involved in regulating stretch-induced tone and L-NAME induced contractions in senescence, notably in the absence of a contractile agonist. Phosphorylation of both NM-II and PAK1 was much more prominent in s-BAs than in y-BAs and was reduced by Y27632 in s-BAs along with the attenuation of stretch-induced tone and L-NAME induced tone. We note that global inhibition of phosphatase activity using calyculin resulted in increased subcortical F-actin (Figure 11). Our findings are in keeping with a previous report that myogenic tone was associated with a small increase in filamentous actin (Moreno-Dominguez et al., 2013). Different from airway smooth muscle, where these phosphorylation events play an important role in agonist induced contraction at young age [reviewed in Zhang et al. (2015)], they appear to be silenced in cerebral arteries in young age, but they are markers of cerebrovascular senescence and intrinsically active, i.e., in the absence of a contractile agonist.

### Caldesmon acts as a molecular brake on stretch-induced tone

In addition to activation of signaling molecules that eventually lead to actin polymerization, PAK1 also works through the actin binding protein caldesmon. Phosphorylation of caldesmon reverses its inhibitory effect on actomyosin MgATPase activity thereby increasing force in skinned smooth muscle at low levels of MLC_20_ phosphorylation (Van Eyk et al., 1998). Technically, it was not possible to monitor PAK1 mediated phosphorylation of caldesmon due to lack of commercial antibodies. Nor was it possible to inhibit caldesmon-phosphorylation by PAK1. Therefore, we used BAs from heterozygous mice, in which the *Cald1* gene was ablated, thus reducing the caldesmon content to ∼50% of WT mice (Figure 9). The decrease in caldesmon level was expected to reduce its inhibitory activity. Old BAs from Cald1^+/−^ mice exhibited stretch-induced tone at an age in which stretch-induced tone was not yet present in WT. Similar to our findings, downregulation of caldesmon in carotid arteries *ex vivo* by siRNA resulted in cross-bridge cycling in unstimulated tissue (Smolock et al., 2009). It is noteworthy that caldesmon immunoreactivity dramatically decreased as a long-term consequence of subarachnoid hemorrhage (Oka et al., 1996; Doi et al., 1997) a status, which is typically associated with a hyper-contractile response of the vasculature. Our finding corroborates previous findings (Smolock et al., 2009; Putz et al., 2021) showing that caldesmon acts as a molecular brake on tone in aged cerebral vessels (Figure 9). Inhibition of caldesmon by phosphorylation, e.g., by upregulation of ROK-PAK1 signaling cascade in senescence, might therefore be a mechanism that leads to a hyper-contractile state in the absence of a contractile stimulus in senescence. Interestingly, myogenic constriction in young rat cerebral arteries was not associated with altered caldesmon phosphorylation, and was proposed not to be involved in tone regulation (Moreno-Dominguez et al., 2014).

Caldesmon, which is expressed in smooth muscle cells of the walls of visceral organs as well as in the vasculature including the medial layer of human cerebral vessels (Kacem et al., 2006), has two important functional domains (Morgan and Gangopadhyay, 2001). Inhibition of actomyosin interaction is exerted by the C-terminal actin-binding domain (Katsuyama et al., 1992; Pfitzer et al., 1993a), while the N-terminal domain binds to myosin and was proposed to be important for positioning caldesmon (Hemric and Chalovich, 1990; Lee et al., 2000). Blocking of myosin binding by inhibitory peptides resulted in an increase in resting tone in permeabilized arteries (Pfitzer et al., 2005). Ablation of exon2 of the *Cald1* gene in mice (CaD-ΔEx2^−/−^), which encodes the strong myosin binding domain, resulted in expression of a truncated caldesmon but the expression level did not differ from WT mice. *In vitro* binding of the truncated caldesmon, isolated from these mice, had lost myosin but not actin binding (Figure 10). However, vascular tone in o-BAs from homozygous CaD-ΔEx2^−/−^ mice was not different from that in WT mice. This indicates that the C-terminal domain is sufficient for inhibition of force.

Taken together, our work corroborated the notion of the central role ROK has as a signaling node that orchestrates several downstream processes, essential for regulating vascular tone both in y- and s-BAs. Our observations expand previous reports that inhibition of ROK reduced vascular tone of pulmonary, cerebral, coronary, and mesenteric arteries from different species including humans, typically performed in young arteries (Fu et al., 1998; Matsumura et al., 2001; Kitazono et al., 2002; Grisk et al., 2012). However, our work suggests that the downstream ROK activated processes differ especially in senescent cerebral arteries from those in young and old ones. We propose that in y-and o-BAs the canonical pathways, i.e., inhibition of MLCP by ROK and increase in phosphorylation of MLC_20,_ predominate tone regulation, while in senescence regulation of tone involves ROK related thin filament linked mechanisms.

### Clinical relevance and perspectives

Several *in vivo* studies in animal models demonstrated that ROK inhibition prevented coronary and cerebral artery vasospasm and neointimal formation after balloon injury of carotid arteries (Kandabashi et al., 2000; Sato et al., 2000; Sawada et al., 2000). However, ROK inhibition may have a number of unwanted side effects. This is because ROK serves as a master regulator in non-muscle cells, regulating such diverse functions as cell-motility, -migration, and -adhesion, thrombocyte aggregation, tumor cell activity as well as the barrier function of endothelial cells (Aslan and McCarty, 2013; Constantin, 2016; Kataoka and Ogawa, 2016; Shimokawa et al., 2016).

1. Therefore, one goal of the present work was to delineate the ROK substrates NM-II and PAK1-phosphorylation as regulators of spontaneous tone generation in the senescent cerebrovascular system. Based on our study, it is reasonable to conduct further studies, investigating whether targeting of these phosphoproteins would be an approach to treat cerebrovascular dysfunction in the elderly.
2. Genetically lowering caldesmon expression leads to early onset of spontaneous contractions in o-BAs, typical for senescence. Therefore, it will be intriguing to investigate, at least in animal models, whether re-expression or overexpression of caldesmon would be able to prevent hypercontractility in cerebral vasculature.

## Data Availability

The authors acknowledge that the data presented in this study must be deposited and made publicly available in an acceptable repository, prior to publication. Frontiers cannot accept a manuscript that does not adhere to our open data policies.
