# Supplementary material for: Dual thick and thin filament linked regulation of stretch- and L-NAME-induced tone in young and senescent murine basilar artery
Source: Front Physiol. 2023 Mar 28;14:1099278. doi: 10.3389/fphys.2023.1099278 (PMC10088910; doi:10.3389/fphys.2023.1099278)
Supplement: Supplementary file 1 [file DataSheet2.docx]

**Dual thick and thin filament linked regulation of stretch- and L-NAME-induced tone in young and senescent murine basilar artery**

Lubomir T. Lubomirov^1,3,7,^*^#^*^,^*, Mechthild Schroeter^1,2,^ *^#^*, Veronika Hasse^1^, Marina Frohn^1^, Doris Metzler^1^, Maria Bust^1^, Galyna Pryymachuk^4,8^, Jürgen Hescheler^2^, Olaf Grisk^3,7^, Joseph M. Chalovich^5^, Neil R. Smyth^6^, Gabriele Pfitzer^1^, and Symeon Papadopoulos^2^

*^1^Institute of Vegetative Physiology, Center of Physiology, University of Cologne, Germany*

*^2^Institute of Neurophysiology, Center of Physiology, University of Cologne, Germany*

*^3^Institute of Physiology, Brandenburg Medical School Theodor Fontane, Germany*

*^4^Institute of Anatomy, University of Cologne, Germany*

*^5^Department of Biochemistry & Molecular Biology, Brody School of Medicine at East Carolina University, Greenville, NC, U.S.A.*

*^6^Biological Sciences, Southampton General Hospital, University of Southampton, Southampton, UK*

*^7^Research Cluster, Molecular Mechanisms of Cardiovascular Diseases, Brandenburg Medical School Theodor Fontane, Neuruppin, Germany*

*^8^Institute of Anatomy,* *Brandenburg Medical School Theodor Fontane, Germany*

*^#^Equal author contribution*

**Author for correspondence*

Short title: Rho-kinase dependent cerebrovascular hypercontractility in senescence

Lubomir T. Lubomirov, PhD

Institute of Physiology

Brandenburg Medical School Theodor Fontane

H 11, 2 OG, R 3.35

Hochstraße 29

14770 Brandenburg an der Havel

Germany

**Supplemental Materials and Methods**

**I. Ethics statement.** All experiments were performed in accordance with the European Community guidelines (Directive 2010/63/EU) and approved by the State Office for Nature, Environment and Customer Protection North Rhine Westphalia: AZ 84-02.05.50.15.029). WT (Wild type): young adult mice (> 2 months; n=29), adult senescent (> 24 months, n=34); heterozygotes T696A/+ (gene point mutation of the targeting subunit of myosin light chain phosphatase, MYPT1 at position 696): young adult mice (> 2 months; n=15), senescent mice (> 24 months, n=29); Cald1 (heterozygous deletion mutation of the Cald1 gene coding for caldesmon protein): adult young mice (> 3 months, n=3) , old mice (> 20 Months, n=14); CaD-ΔEx2^−/−^ (homozygous mice with deleted strong myosin binding site of caldesmon, originally generated at Institute of Physiology, University of Cologne): old mice (> 20 months,; n=7), respective wild type littermates (n=7) for experiments with CaD-ΔEx2 were purchased from Center for Molecular Medicine Cologne (ZMMK) and randomly allocated to the different sets of experiments. All experiments were planed and performed in compliance with the ARRIVE guidelines (https://www.nc3rs.org.uk/arrive-guidelines).

**II. Generation of CaldesmonΔEx2^-/-^ mice, biochemical verification and binding assays to myosin and actin of the truncated caldesmon protein**.

*Generation of Caldesmon* Δ*Ex2^-/-^ mice.* Exon2 of the *Cald1-*gene was targeted by homologous recombination that led to disruption of exon2, which codes for the strong myosin binding site of caldesmon (Pfitzer et al. 2005). Briefly, the 5`arm of the targeting vector was composed of a KpnI-ApaI 2.3kb homology region, containing part of intron 1 and the first 63 base pairs of exon2. The 3’arm was formed by an 8kb PstI-HindIII homology region, spanning from intron 2 to intron 4. Both vector arms were cloned in pBS°II°KSII (Stratagene). As resistance cassette, a PGK-Neo was inserted using the NotI site of the vector. The E14 embryonic stem (ES) cell subline, IB10, was transfected by electroporation with 25µg of Sal I linearized construct and selected with 400 µg/mL G418. Resistant clones were isolated and analyzed for correct insertion by Southern blotting and probing with two external (5` in intron 1`; 3` in intron 4) and one internal probe (in neomycin cassette). ES cells from two independent heterozygous cell clones were injected into C57/Bl6 blastocysts and resulting chimeric male offspring were mated with wild type C57BL/6 mice. Germ line transmission was identified by Southern blot analysis of progeny mouse tail biopsies (Suppl. Figure 1). Heterozygous mice were bred to the original C57BL/6 strain for at least nine generations to produce congenic C57BL/6 background. Heterozygous mice were then mated to produce homozygous mutant mice. All breeding steps were controlled by Southern blot analysis or PCR (Suppl. Figure 1).

*Sequence analysis of mouse caldesmon cDNA.* Deletion of Exon2 in both caldesmon isoforms (high and low, (Hayashi et al. 1992)) was confirmed by sequence analysis of cDNAs generated by reverse transcription of RNAs isolated from fibroblasts (l-CaD) and urinary bladders (h-CaD) of mutant mice (RNAeasy Mini Kit, QIAGEN GmbH, Hilden, Germany). To generate caldesmon cDNA, primers (5` AGCAAGCTTGCCGCCACGATGGATGATTTTGAACGTCGCAG 3` and 5` GCAAGCTTATGCTTAGCGGATCCGGGTCC 3`) from exon1 and exon1` respectively and a reverse primer (5` TAGCGGCCGCGACCTTAGTGGGGGAAGTGAC 3`) from exon13 for both caldesmon isoforms were used. PCR products were subcloned in pBS II KS using NotI and HindIII digestion sites included in the primers and fully sequenced.

[https://www.ncbi.nlm.nih.gov/nuccore/OQ623826 [ncbi.nlm.nih.gov]](https://urldefense.com/v3/__https:/www.ncbi.nlm.nih.gov/nuccore/OQ623826__;!!OTyOlAGPvQ!WDVyKkMLHP1D8yek2nKEqbpxkmLzNSDvbZqw0F5q8jPhkGGLC23nck39XkO9a4MDrwNB5BsOX10_WK3DeeIKw4ZbJjA-jZUQz7aekK8SPgLmYg$)

[https://www.ncbi.nlm.nih.gov/nuccore/OQ623827 [ncbi.nlm.nih.gov]](https://urldefense.com/v3/__https:/www.ncbi.nlm.nih.gov/nuccore/OQ623827__;!!OTyOlAGPvQ!WDVyKkMLHP1D8yek2nKEqbpxkmLzNSDvbZqw0F5q8jPhkGGLC23nck39XkO9a4MDrwNB5BsOX10_WK3DeeIKw4ZbJjA-jZUQz7aekK9dmRgWqQ$)

*Analysis of specific peptides by peptide mass fingerprint (MALDI-TOF)*. Urinary bladders of WT and mutant mice were removed immediately after euthanization and shock-frozen in liquid nitrogen. Tissues were homogenized in 300µl of FOCUS^TM^/Protein Solubilization Buffer (GenoTech, St. Louis, MO, USA), sonicated for 6 min and centrifuged at 20.000g for 30 min at 4°C. Proteins of the supernatants were separated by 2D-PAGE. The gels were Coomassie stained. CaD spots were identified by Western blotting with a polyclonal antibody raised in rabbits against chicken gizzard caldesmon (dilution 1:1000). A peptide fingerprint analysis was made of the CaD spots. Positive ion spectra were acquired on a Reflex IV MALDI-TOF mass spectrometer (Bruker Daltonics, Bremen, Germany) in the reflectron mode. XMASS 5.1.10 software was used for internal recalibration on trypsin autolysis peaks and the generation of peak-lists. Protein identity was confirmed by comparison with the NCBInr public database (National Center for Biotechnology Information) using MASCOT 1.9.

*Protein purification.* Caldesmon was isolated from 2.4 g of shock-frozen urinary mouse bladders prepared by a modification of the method of Bretscher (Bretscher 1984). Myosin was prepared from rat bladder tissue (Persechini and Hartshorne 1983). Calmodulin was prepared from wheat germ (Anderson 1983). Other proteins were prepared as described elsewhere (Schroeter and Chalovich 2005).

*Binding of caldesmon to myosin in a co-sedimentation-assay.* Smooth muscle myosin (0.5 µmol/L) from rat was mixed with CaD in a 0.51 M ionic strength solution. The mixtures were dialyzed against (in mM) 30 NaCl, 10 MOPS (pH 7.0), 5 MgCl_2_, 1 EGTA, and 1.5 dithiothreitol. ATP was added to 0.2 mL aliquots of each mixture to a final concentration of 1 mmol/L. Samples were centrifuged for 5 minutes at 19,000 rpm in a TLA 120.1 rotor at 10°C.  Pellets were analyzed by SDS polyacrylamide gelelectrophoresis. Gels were analyzed as described earlier (Fredricksen et al. 2003).

*High speed centrifugation assay of F-actin binding.* CaD samples were freshly dialyzed against (in mM) 10 imidazole (pH 7.0), 100 NaCl, 1 EDTA, 1 EGTA, and 2 dithiothreitol.  1 µmol/L wild type or truncated caldesmon was mixed with 5 µmol/L F-actin in a total volume of 0.2 mL, incubated on ice for 1 h and centrifuged for 1 h at 35,000 rpm in a TLA 120.1 rotor. Pellets were swollen in 20 µL H_2_O for 1 h and processed for electrophoresis as described in the previous section.

**III. Tissue Preparation.** All animals were anesthetized with isoflurane and sacrificed by decapitation. Brains were removed, transferred to Petri-dishes, filled with ice cold HEPES-buffered low Ca^2+^ physiological saline solution (HPSS, see solutions), and the basilar arteries were dissected. Preparations were cut out into two pieces with approx. 1.9 mm length and kept for 10 minutes in the same solution. Thereafter, arterial rings were mounted in a wire-myograph (model 610A, Danish Myotechnology, Aarhus, Denmark) in HPSS and kept for equilibration for another 10 minutes. Then, HPSS was replaced by carbogen (95% O_2_ / 5% CO_2_)-aerated bicarbonate buffered solution (PSS, see solutions) and temperature was slowly increased to 37°C. Thereafter, the ring preparations were stretched stepwise, until 90% of the internal circumference was reached and the wall tension corresponded to a transmural pressure of 100 mmHg (IC90), as described previously (Mulvany and Halpern 1977). In the organ bath, all substances were added directly and pH was kept at 7.4 during the whole experiment.

**IV. Contractile protocols**

**1. Measurement of stretch-induced–tone and concentration-response relationships to thromboxaneA2-analogue U46619 in young and senescent basilar arteries** **from WT, MYPT1-T696A/+, Cald1+/-, and CaD-ΔEx2−/− mice**

After pre-stretching, preparations of basilar arteries were equilibrated for 20-30 minutes in PSS until they developed stable tone, latter denoted in this work as stretch-induced-tone. Then, endogenous NO-release was inhibited by the pan-NOS-inhibitor N^G^-Nitro-L-arginine methyl ester (L-NAME) 100 µmol/L. Thereafter, arteries were stimulated by cumulative application of stable thromboxaneA2 analogue, U46619, starting with 0.003 µmol/L up to a final concentration of 3 µmol/L. After force reached maximum, arteries were washed out several times with PSS and incubated with 2 mmol/L EGTA in PSS to obtain vascular tone under Ca^2+^-free conditions.

**2. Stretch-induced–tone and concentration relationship of U46619 in young basilar arteries from MYPT1-T696A/+ and WT mice**

This stimulation protocol differs slightly from the previously described protocol. In brief, after pre-stretching and 20-30 minutes equilibration, preparations of y-BAs (> 2 month) from MYPT1-T696A/+ and WT mice, were cumulatively stimulated with U46619 (conc. range 0.003-3 µmol/L). Then arteries were washed out several times with PSS until tone recovered. Thereafter, endogenous NO-release was inhibited by application of 100 µmol/L L-NAME for 20 minutes and a second concentration-response curve was obtained. Finally, basal vascular tone was determined under Ca^2+^-free conditions by washing preparations several times with PSS and application of 2 mmol/L EGTA into the organ bath of the myograph.

**3. Ca^2+^- responsiveness of α-toxin permeabilized y-BAs from WT and MYPT1-T696A/+ mice**

In these experiments, after isolation in HPSS, y-BAs from WT and MYPT1-T696A/+ mice were placed for 15-20 minutes in 500 µL reaction tubes, filled with 250 µl HPSS with 2 mmol/L EGTA (Ca^2+^-free). Thereafter, arteries were placed in a new reaction tube containing relaxing solution (10 mmol/L EGTA; see solution), pCa >8. After incubation for further 15 minutes, arteries were permeabilized with staphylococcus aureus α-toxin (conc. 40 Units pro µL; final volume 50 µL) in relaxing solution. Finally, arterial rings were mounted in relaxing solution on 25 µm tungsten wires in a myograph (model 610A) under continuous air bubbling and subjected to normalization as described above. To deplete Ca^2+^-stores after normalization, arteries were treated with the Ca^2+^-ionophor A23178, 10 µmol/L, for 20 minutes Thereafter, the relaxing solution was replaced by solutions with increasing pCa (7.0, 6.6, 6.2, 5.8 and 4.3), 10 minutes each. After force reached maximum at pCa 4.3 at the end of the experiment, preparations were relaxed in solution with pCa>8 (relaxing solution) for 20-30 minutes. Unless indicated otherwise, experiments were carried out at room temperature, 23°C.

**V. Protein extraction and immunoblotting**

BAs from each mouse strain were isolated in PSS as described above and mounted on 25 µm tungsten wire. The wires were placed in PSS-filled 1.5 mL reaction tubes and equilibrated for10 minutes under constant aeration with carbogen (95% O_2_ / 5% CO_2_). Then reaction tubes were incubated at 37°C for 30 minutes. Thereafter, preparations were treated for 10 minutes with either 3 µmol/L Y27632, or 10 µmol/L blebbistatin(+), or 10 µmol/L blebbistatin(-), or vehicle (H_2_O or DMSO, 0.3% final volume). Finally, BAs were incubated for 15 minutes with 100 µmol/L L-NAME. The preparations were then shock-frozen in 15% trichloro-acetic acid/acetone (TCA/acetone) slurry, precooled with dry ice, and fixed at -80°C for 3h or overnight. TCA was removed by rinsing the preparations with acetone on dry ice. Specimens were dried for 10 minutes at room temperature. Specimens were homogenized in 40 µL Laemmli buffer (see solutions and buffers) using tissue grinders (Kimble Chase LLC, Tissue Grinder Micro PKG/6, USA) and extracted for 1 h on ice. Afterwards, tissue homogenates were centrifuged at 20,000 g and 4°C and the supernatant was subjected to SDS-PAGE (gradient gels with 4-20% acrylamide). Proteins were transferred overnight to nitrocellulose membranes (Amersham Biosciences Europe GmbH) in Towbin-transfer-buffer (SERVA electrophoresis GmbH), at 4°C and constant voltage, 22 V. After transfer, membranes were rinsed twice with H_2_O and the quality of the protein transfer was controlled by visualization of the protein bands using Ponceau-S. Then, the membranes were incubated with respective primary and secondary antibodies (see paragraph “Antibodies and antibody dilutions”) for 4-5 h and 1 h respectively at room temperature. Immunoreactive signals were visualized using the Odyssey Infrared Imaging System (LI-COR Inc.) or enhanced chemiluminescence (West Pico, Thermo Fisher Scientific (Waltham, MA, USA).

**Antibodies and antibody dilutions:** anti-phospho-MLC_20_-S19 (pMLC_20_-S19), rabbit polyclonal, dilution 1:1,500 (Rockland Immunochemicals Inc. Limerick, PA, USA); anti-phospho-MYPT1-T853 (pMYPT1-T853) rabbit polyclonal, dilution 1:4,000 (Merck KGaA/Millipore, Darmstadt, Germany); anti-phospho-MYPT1-T696 (pMYPT1-T696), rabbit polyclonal, dilution 1:50,000 (Merck KGaA/Millipore); anti-MYPT1-total, mouse polyclonal, dilution 1:5,000 (BD Transduction Laboratories, Franklin Lakes, NJ, USA); anti-α-actin, mouse monoclonal, dilution 1:50,000 (Sigma-Aldrich/Merck); anti-GAPDH, rabbit polyclonal, dilution 1:10,000 (Sigma-Aldrich/Merck); SM-22, goat monoclonal, dilution 1:20,000 (Novus Biologicals/ Bio-Techne); anti-phospho-Myosin IIa-Ser1943 dilution 1:500 from Cell Signaling Technology, (Inc. Danvers, MA, USA); rabbit polyclonal from anti-Myosin IIa, dilution 1:2,000 (Cell Signaling Technology, Inc. Danvers, MA, USA); horseradish peroxidase coupled donkey anti-rabbit secondary antibody, dilution 1:10,000 (Jackson ImmunoResearch Laboratories Inc., (DIANOVA)); goat anti-mouse IG DyLight 680 and goat anti-rabbit IG DyLight 800 dilution 1:10,000(Thermo Fisher Scientific, Waltham, MA, USA).

**VI. Determination globular/ fibrillar actin ratio (G/F-actin)**

The G/F-actin was determined with the G-actin/F-actin *in vivo* Assay Kit BK037 according to the manufacturer’s instructions (Cytoskeleton, Inc. / Biomol). In brief, BAs of WT and T696A/+ senescent animals (> 24 months) were prepared as described above and the whole BA with an approximate length of 3.8 mm was isolated in HPSS and immediately shock-frozen in liquid N_2_. Specimens were transferred in 37°C pre-warmed tissue grinders, containing 60 µL “Lysis and F-actin Stabilization Buffer”, and homogenized at the same temperature. Homogenates were transferred in 1.5 mL Eppendorf® Safe-lock reaction tubes and centrifuged at 350 g, at 37°C for 5 minutes. In order to separate F-actin from G-actin, the supernatants were gently transferred into Beckman Coulter Microfuge® tubes and centrifuged for 1 h at 100,000 g and 37°C using a TLA-55 Fixed-Angle-rotor (Beckman). The G-actin containing supernatant was carefully removed and transferred in a fresh reaction tube. The F-actin containing pellet was dissolved in “F-actin Depolymerizing Buffer” and incubated on ice for 1 h. To help resuspension, the pellet was gently agitated every 10 minutes. After the pellet was completely dissolved, 2x Laemmli-sample buffer was added to both, the supernatant and the pellet, to give a final concentration of 1x Laemmli-Sample-Buffer. Samples were then subjected to SDS-PAGE and immunoblotting as described before. Immunoreactive signals were detected using polyclonal rabbit anti-pan-actin antibody (1:10,000) supplied with the kit.

**VII. Visualization of fibrillar actin in y- and s-BAs using confocal microscopy**

BAs with equal dimensions from young and senescent WT-animals were isolated, mounted in the myograph and normalized as described previously. After equilibration for 20 minutes at IC90, the arteries were stimulated for 30 minutes with 0.1 µmol/L calyculinA, which slowly increased the tone. Then, the arteries were fixed in the myograph for 45 minutes in 4% paraformaldehyde in PBS at room temperature while shaking gently. Thereafter, preparations were permeabilized with 1% TritonX-100 in PBS for 45 minutes followed by 6 washing steps with PBS. Specimens were gently removed from the wires, placed on cover glasses and dried completely. Preparations were reconstituted in PBS and non-specific binding sites were blocked with 3% bovine serum albumin while shaking gently. F-actin and nuclei were simultaneously stained for 1 h with Alexa Fluor^TM^ 555-phalloidin (1:5,000) and Hoechst 33342 (1:1,000) in PBS. Finally, to remove unbound dye, specimens were washed 5 times for (altogether) 20 minutes (each washing step) and mounted on slides by applying Aqua Poly/Mount solution (Polysciences Inc USA). Structures were visualized using a confocal microscope Olympus Fluoview1000 (60X/oil magnification). Nuclei were visualized via the 440 nm line of a blue diode laser (465-495 band pass filter) with marginal, but sufficiently high excitation of Hoechst at the long-wavelength border of its excitation spectrum. Alexa Fluor^TM^ 555 conjugated phalloidin stained F-actin was visualized via the 543 nm line of an argon ion laser (560 nm long pass filter). Intensity of the excitation beams was kept constant throughout the measurements. Slices (18-22) varied according to the vessel thickness were recorded in the x-y-z stack confocal mode, while keeping the z-steps constant at 1 µm. The dimensions of the x-y scans were 512x512 pixel, corresponding to 1 µm. Z-stacks were recorded from at least three different regions of each preparation.

**VIII. Chemicals, solutions, and buffers**

**1. Chemicals**

N^ω^-Nitro-L-arginine methyl ester hydrochloride (L-NAME), Y27632 (Merck KGaA, Darmstadt, Germany); U46619 (TOCRIS Bioscience, Abingdon, United Kingdom); blebbistatin (+) and blebbistatin (-) from (Sigma-Aldrich/Merck); calyculin A (Cell Signaling Technology, Inc. Danvers, MA, USA).

**2. Solutions for wire myography**

***HEPES buffered physiological salt solution with low Ca^2+^ (HPSS) in mmol/L***: 118 NaCl, 5 KCl, 1.2 NaH_2_PO_4_, 1.2 MgCl_2_, 0.16 CaCl_2_, 10 Glucose, and 24 HEPES, pH 7.4 at room temperature.

***Physiological salt solution (PSS; in mmol/L*):** 119 NaCl, 4.7 KCl, 1.18 KH_2_PO_4_, 1.17 MgSO_4_, 1.6 CaCl_2_, 5.5 glucose, 25 NaHCO_3_, 0.03 EGTA, pH 7.4 at 37°C, equilibrated with carbogen (95% O_2_, 5% CO_2_).

***Relaxing solution (pCa = −log10[Ca^2+^] ≥ 8; in mmol/L):*** 20 imidazole, 7.5 Na_2_ATP, 10 EGTA, 10 Mg [CH_3_COO]_2_, 10 creatine phosphate, 31.25 potassium methanesulfonate, 5 NaN_3_, 0.01 GTP, 0.001 leupeptin, 2 dithiothreitol; рН 7.00 was adjusted with CaCl_2_-free KOH. ***Contraction solution (pCa 4.3 = −log10[Ca^2+^] = 4.3)*:** This solution had the same composition as the relaxing solution except that it contained 10 mmol/L CaCl_2_.

The solutions with intermediate pCa concentrations (7.0, 6.6, 6.2, 5.8) were obtained by mixing relaxing and contracting solutions in the appropriate ratio (Welter et al. 2020). The ionic strength of the relaxing and contracting solutions were adjusted with potassium methanesulfonate to 150 mmol/L.

**3. Solutions and buffers for Western blotting**

Towbin-transfer-buffer: 25 mmol/L Tris, 0.192 mol/L Glycine, 10% Methanol, 0.05 or 0.005% SDS; Tris-buffered saline solution with Tween20 (TBST) in mmol/L: 20 Tris, 150 NaCl, 0.05 % Tween20; pH 7.6 at room temperature, 23°C; 2x Laemmli-sample-buffer for SDS-PAGE: 126 mmol/L Tris-HCl (pH 6.8), 20 % glycerin, 4 % SDS, and 0,02 % bromophenol blue20 mmol/L dithiothreitol; SERVA Tris-Glycin/SDS-Electrophoresis-Buffer (10x): 250 mmol/L Tris, 1.92 mol/L Glycine, 1 % SDS.

If not indicated otherwise, all chemicals used for SDS-PAGE and Western blotting were purchased by SERVA Electrophoresis GmbH, Heidelberg, Germany.

**IX. Statistics**

Results are given as mean ± SEM. The values of F_max_ are given as absolute force in (mN). If not indicated otherwise, **n** represents the number of animals. The significance level was set at p<0.05 and was tested with Student’s t-test if two pairs were compared or with 2 way-ANOVA for multiple comparisons followed by Tukey multiple comparisons post-test.

**Supplemental Figures**


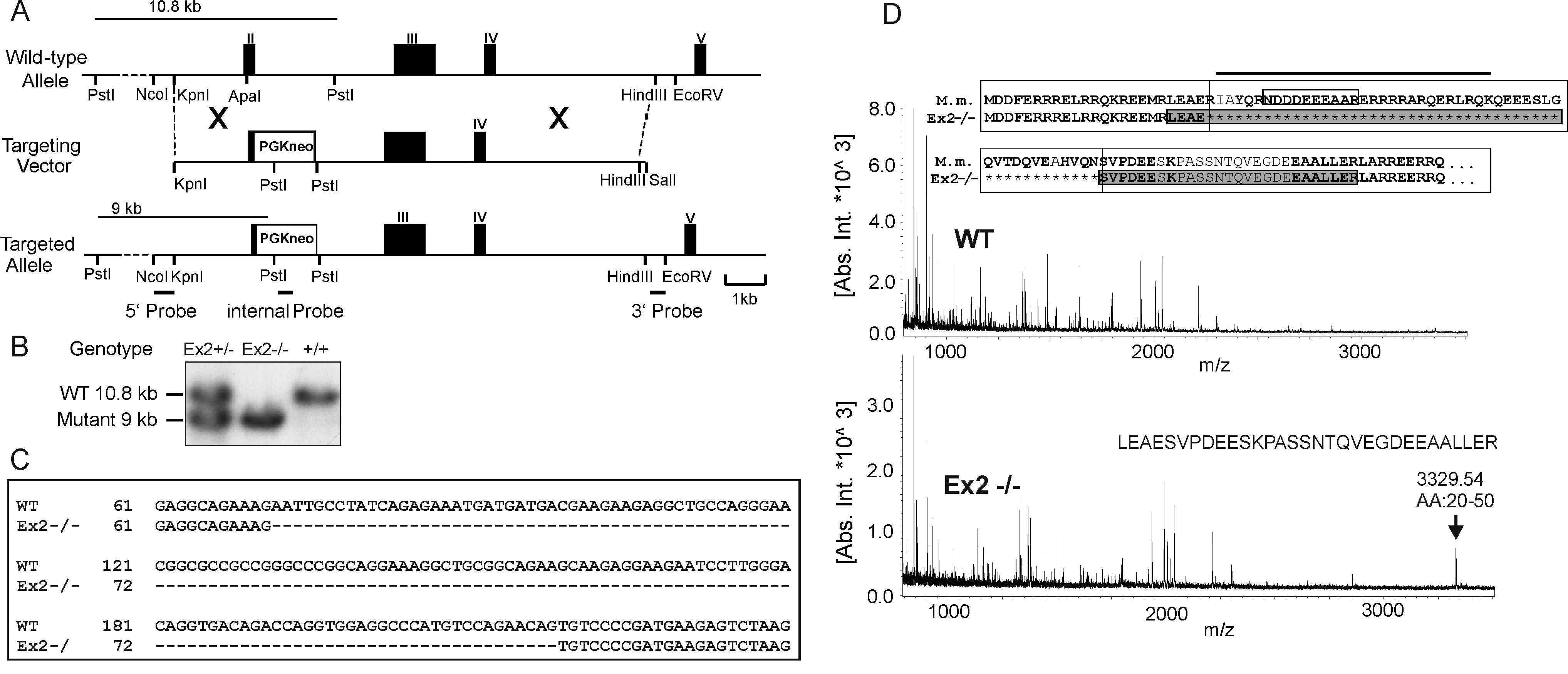


**Suppl. Figure 1. Targeting of exon2 of the CaD gene in the mouse, CaD expression and MALDI-TOF analysis.**

**(A)** Construction of the targeting vector: exon2 was replaced by a neomycin resistance cassette. **(B)** The deletion of exon2 was confirmed by Southern blot analysis of mouse tail DNA, using an external 5`probe (see (A)). **C)** Comparison of WT (GenBank accession number OQ62826) and mutant sequence (GenBank accession number OQ62827); mRNA isolated from urinary bladder was transcribed into cDNA and subcloned for sequence analysis. **(D)** Peptide mass fingerprint analysis and sequence alignment of the predicted amino acid sequence from the mutant mice with the sequence from mouse (M. m.) (inset). The horizontal bar indicates the strong myosin binding site. The tryptic digestion generates specific peptides for the WT and the ΔEx2-/- CaD proteins, marked by a white and grey box respectively in the amino acid sequence (inset). The specific peptide for the mutant protein spans from exon1 to exon3.

**
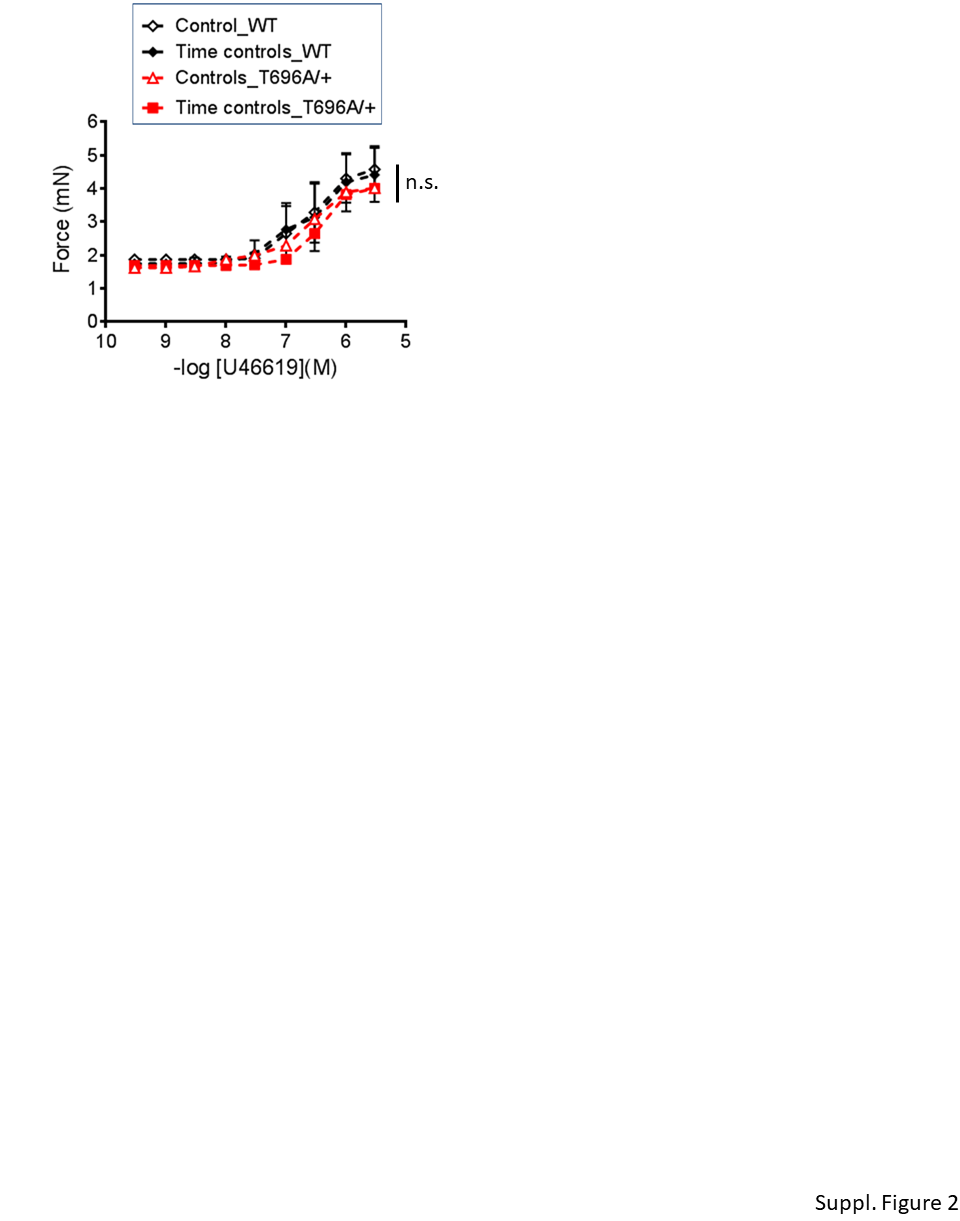
**

**Suppl. Figure 2. U46619-induced tone in y-BA from wild type and MYPT1-T696A/+ mice**

Statistic evaluation of U46619-induced tone in WT and MYPT1-T696A/+ y-BAs (n=7-6). Stimulation protocol: Vessels were first stimulated by increasing concentrations U46619 (controls), washed with PSS and after 20-25 minutes tone recovery challenged again using the same stimulation protocol (Time controls). Data presented as absolute force in mN. n.s -p>0.05; result of statistic comparison of dose responsiveness and absolute force; 2way ANOVA

**
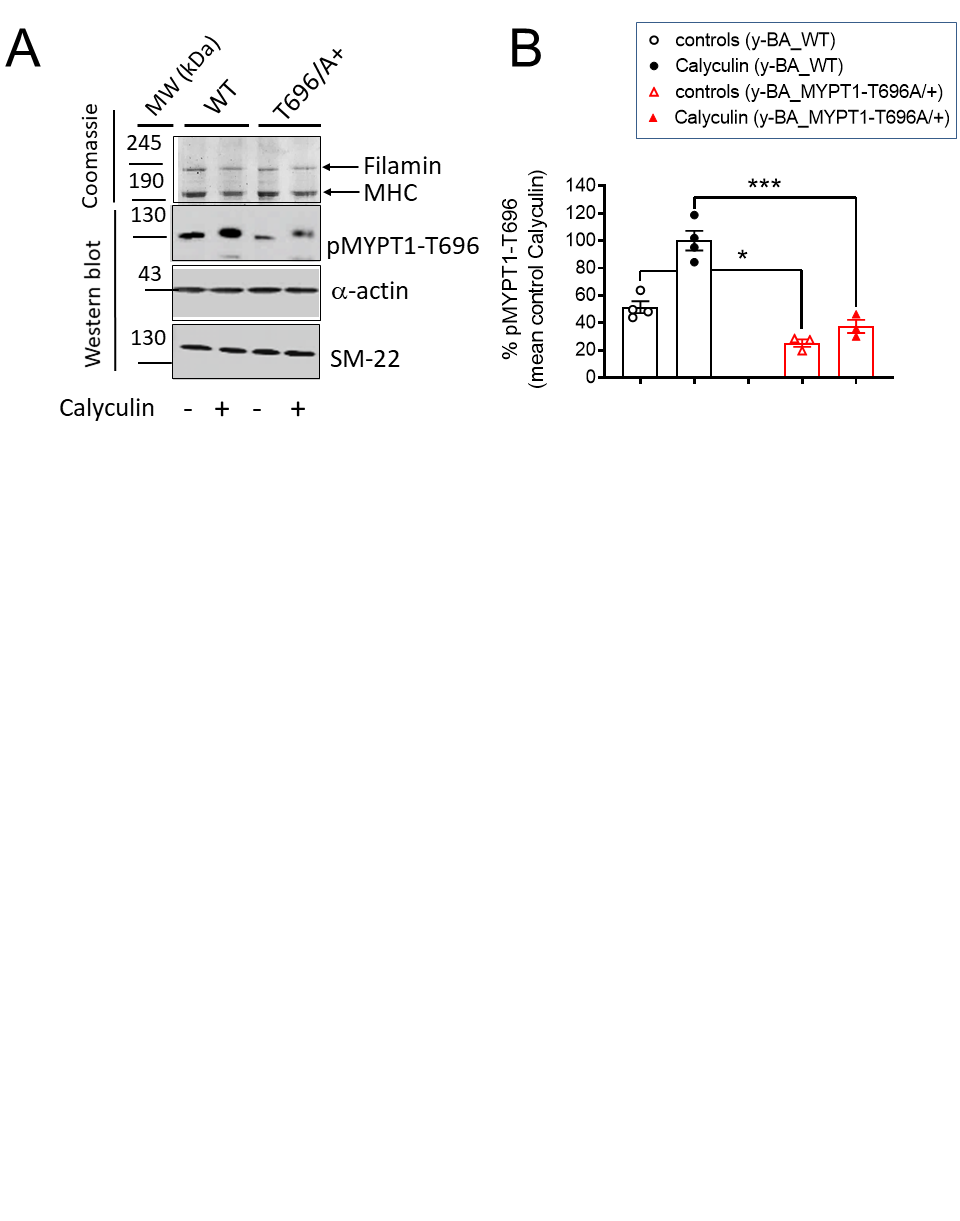
**

**Suppl. Figure 3. Phosphorylation of MYPT1-T696 under inhibition of phosphatases from type II**

**(A)** Original Western blot and **(B)** statistic summary of the phosphorylation of MYPT1-T696 in y-BA from WT and MYPT1-T696A/+ -mice after treatment with the type II phosphatase inhibitor calyculin A. Preparations were treated either with vehicle (0.3% DMSO) or 0.1 µmol/L calyculin A for 30 minutes 3 µmol/L Y27632, shock-frozen and subjected to Western blot as described in “Methods”. *** p<0.001 and *p<0.05 unpaired *t-test*

**
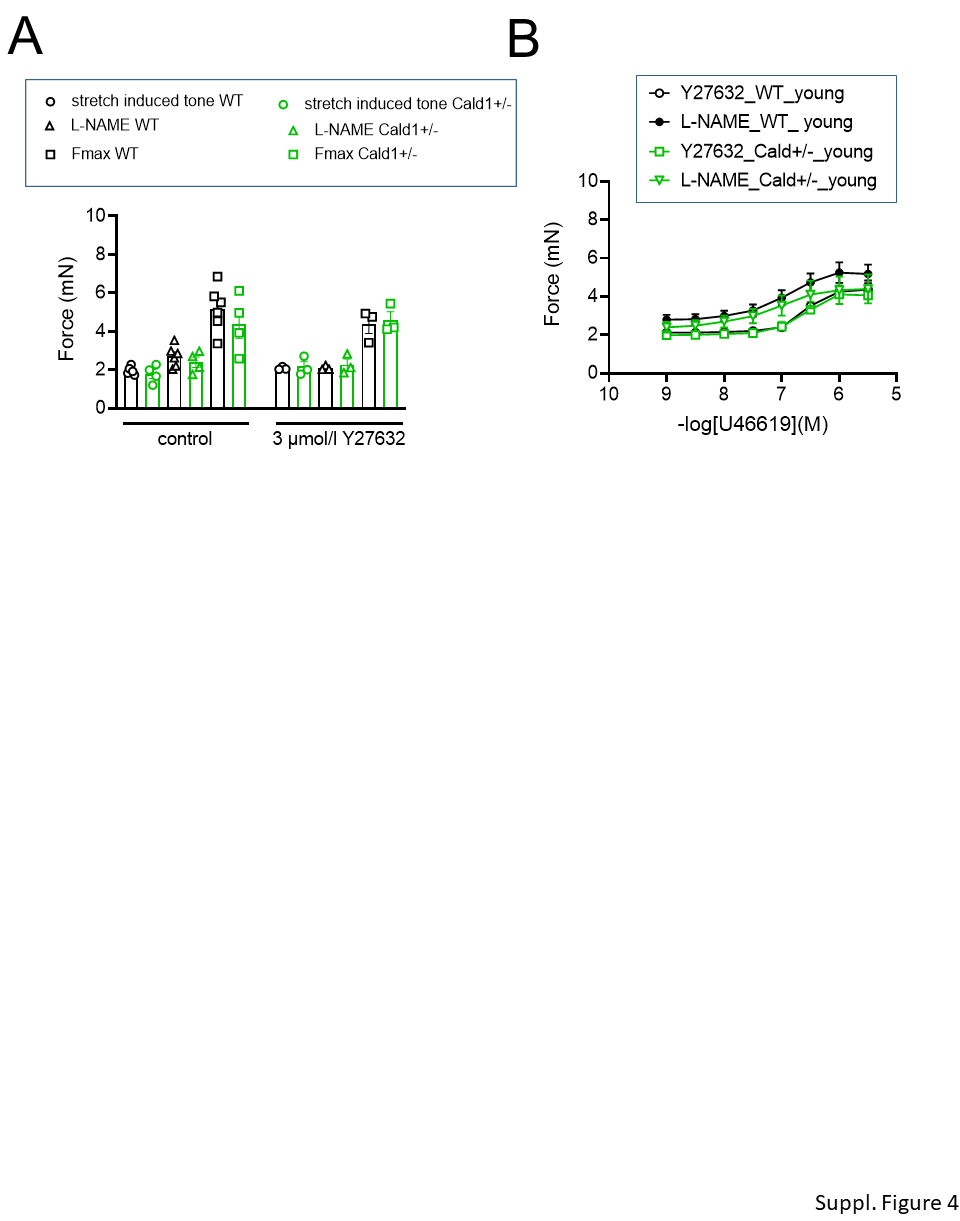
**

**Suppl. Figure 4. Effect of caldesmon targeting on tone in y-BAs**

**(A)** Statistic evaluation of stretch-induced, L-NAME-induced and maximal tone in BAs from young WT and Cald1+/- BAs (n=3). **(B)** Statistic evaluation of tone induced by cumulative application of U46619 (n=3)**.** Data represented as absolute force ± SEM.


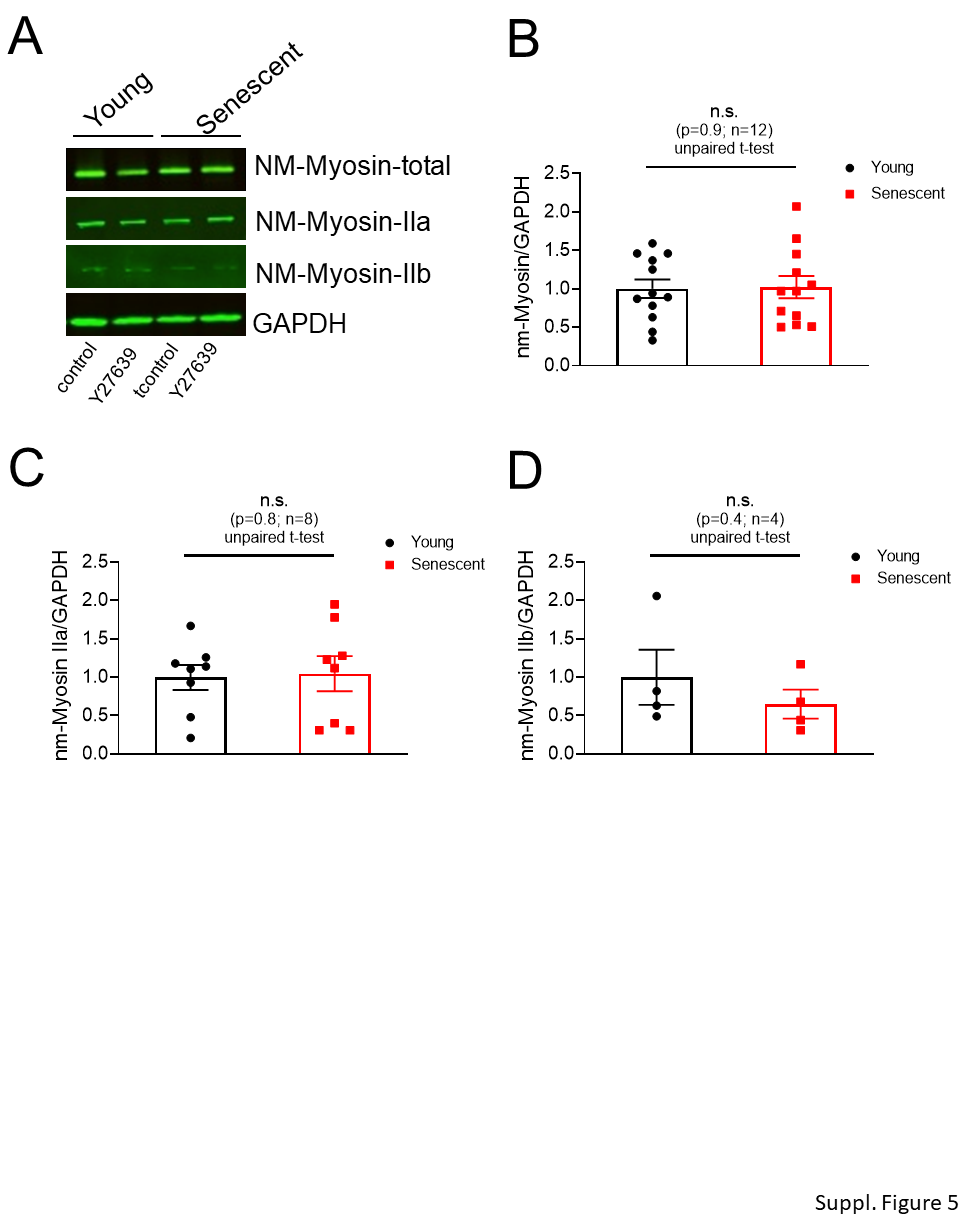


**Suppl. Figure 5. Expression of total, IIa and IIb isoforms of non-muscle-myosin II (NM-II) in y- and s-BAs**

**A:** Original Western blots and **(B)** statistic evaluation of the expression of NM-II-total in y- and s-BAs. **(C, D)** Statistic evaluation of the expression of total NM-IIa (C) and total NM-IIb (D). Data represented as ratio between NM-II-total vs. GAPDH, or NM-IIa vs. GAPDH, or NM-IIb vs. GAPDH, normalized to the signal obtained in y-BAs.

**REFERENCES**

Anderson JM (1983) Purification of plant calmodulin. Methods Enzymol 102:9-17 doi:10.1016/s0076-6879(83)02004-2

Bretscher A (1984) Smooth muscle caldesmon. Rapid purification and F-actin cross-linking properties. J Biol Chem 259(20):12873-80

Fredricksen S, Cai A, Gafurov B, Resetar A, Chalovich JM (2003) Influence of ionic strength, actin state, and caldesmon construct size on the number of actin monomers in a caldesmon binding site. Biochemistry 42(20):6136-48 doi:10.1021/bi0274017

Hayashi K, Yano H, Hashida T, et al. (1992) Genomic structure of the human caldesmon gene. Proc Natl Acad Sci U S A 89(24):12122-6 doi:10.1073/pnas.89.24.12122

Mulvany MJ, Halpern W (1977) Contractile properties of small arterial resistance vessels in spontaneously hypertensive and normotensive rats. Circ Res 41(1):19-26 doi:10.1161/01.res.41.1.19

Persechini A, Hartshorne DJ (1983) Ordered phosphorylation of the two 20 000 molecular weight light chains of smooth muscle myosin. Biochemistry 22(2):470-6 doi:10.1021/bi00271a033

Pfitzer G, Schroeter M, Hasse V, et al. (2005) Is myosin phosphorylation sufficient to regulate smooth muscle contraction? Adv Exp Med Biol 565:319-28; discussion 328, 405-15 doi:10.1007/0-387-24990-7_24

Schroeter MM, Chalovich JM (2005) Fesselin binds to actin and myosin and inhibits actin-activated ATPase activity. J Muscle Res Cell Motil 26(4-5):183-9 doi:10.1007/s10974-005-9009-6

Welter J, Pfitzer G, Grisk O, Hescheler J, Lubomirov LT (2020) Epac-mediated relaxation in murine basilar arteries depends on membrane permeability of cyclic nucleotide analogues and endothelial aging. Gen Physiol Biophys 39(2):157-168 doi:10.4149/gpb_2019055
